# Supplementary material for: ALYREF condensation stabilizes m5C-modified PARP10 mRNA and promotes PI3K-AKT signaling in ovarian cancer
Source: EMBO J. 2025 Dec 1;45(2):471–503. doi: 10.1038/s44318-025-00657-0 (PMC12811383; doi:10.1038/s44318-025-00657-0)
Supplement: Supplementary file 9 — Movie EV2 [file 44318_2025_657_MOESM9_ESM.zip › Movie EV2/Legends of EV2.docx]

**Movie EV2**. FRAP assays detecting the fluorescence signal recovery of a tGFP-ALYREF fusion protein after bleaching in A2780 cells.
