# Supplementary figures and images for: ALYREF condensation stabilizes m5C-modified PARP10 mRNA and promotes PI3K-AKT signaling in ovarian cancer
Source: EMBO J. 2025 Dec 1;45(2):471–503. doi: 10.1038/s44318-025-00657-0 (PMC12811383; doi:10.1038/s44318-025-00657-0)

## Slide 1
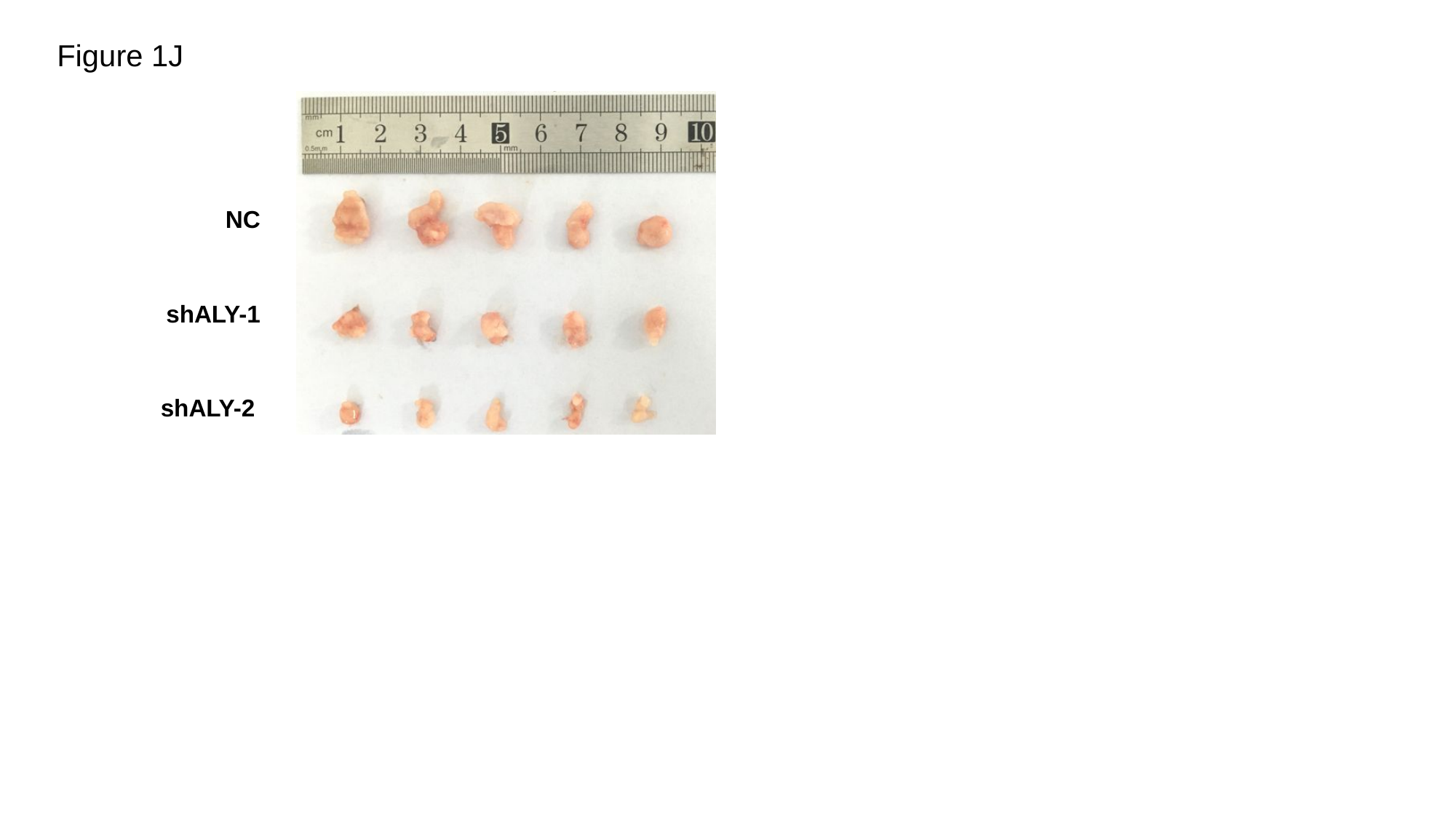

Figure 1J
NC
shALY-1
shALY-2

Supplement: Supplementary file 11 — Source data Fig. 1 [file 44318_2025_657_MOESM11_ESM.zip › Source Data for Figure 1/Source Data for Figure 1J.pptx]

## Slide 1
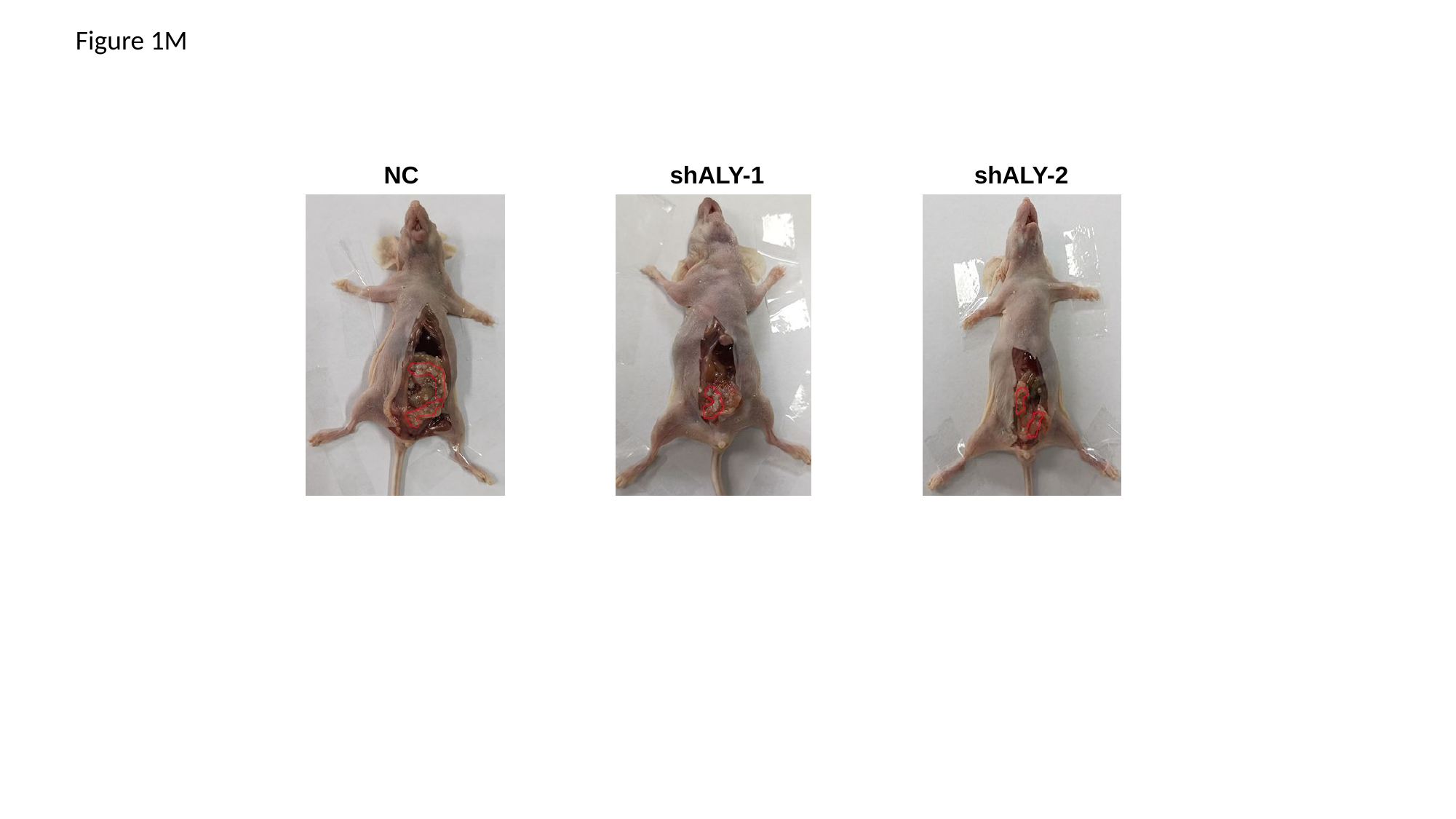

Figure 1M
NC
shALY-1
shALY-2

Supplement: Supplementary file 11 — Source data Fig. 1 [file 44318_2025_657_MOESM11_ESM.zip › Source Data for Figure 1/Source Date for Figure 1M.pptx]

## Slide 1
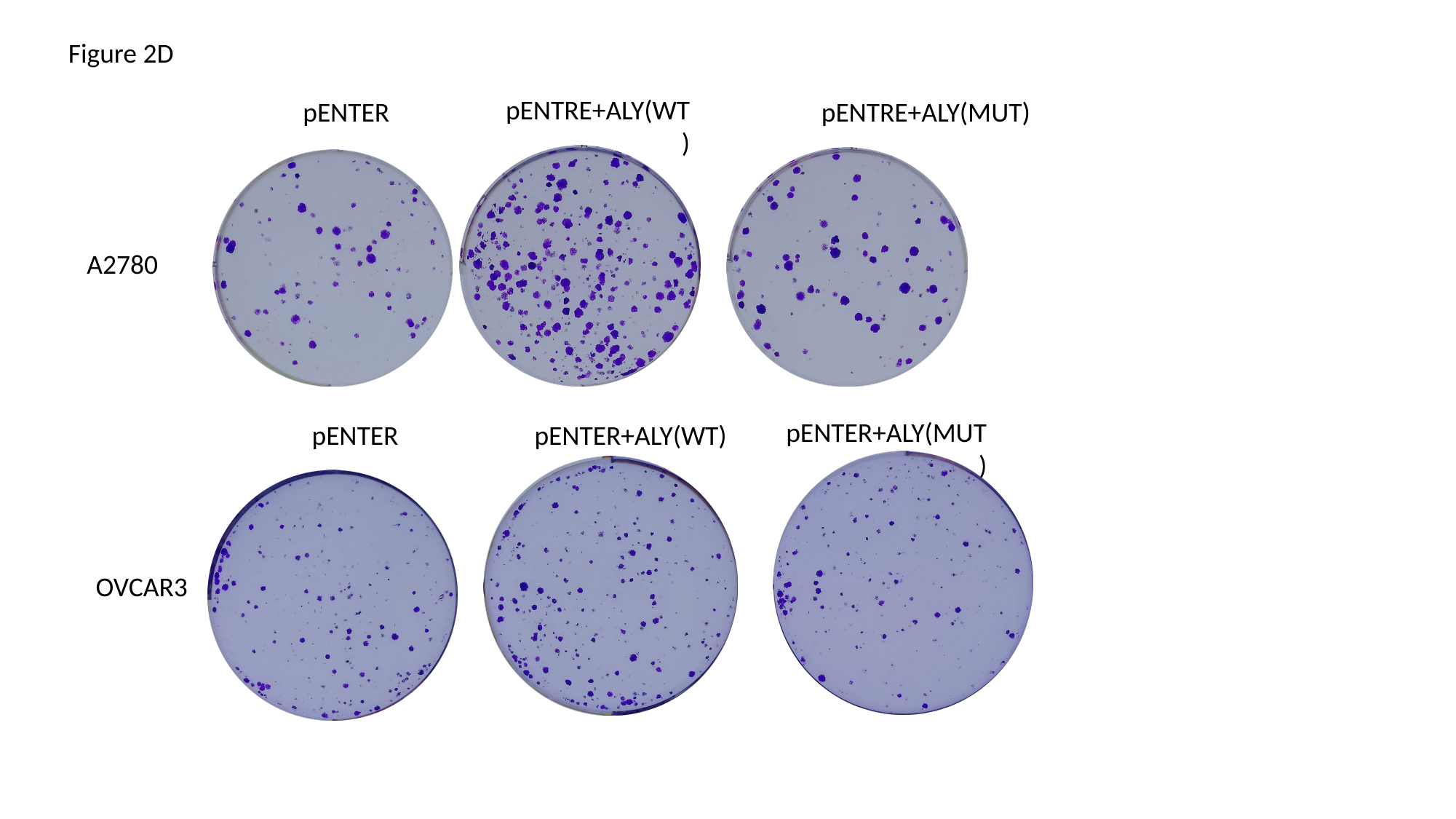

Figure 2D
pENTRE+ALY(WT)
pENTER
pENTRE+ALY(MUT)
A2780
pENTER+ALY(MUT)
pENTER
pENTER+ALY(WT)
OVCAR3

Supplement: Supplementary file 12 — Source data Fig. 2 [file 44318_2025_657_MOESM12_ESM.zip › Source Data for Figure 2/Source Date for Figure 2D.pptx]

## Slide 1
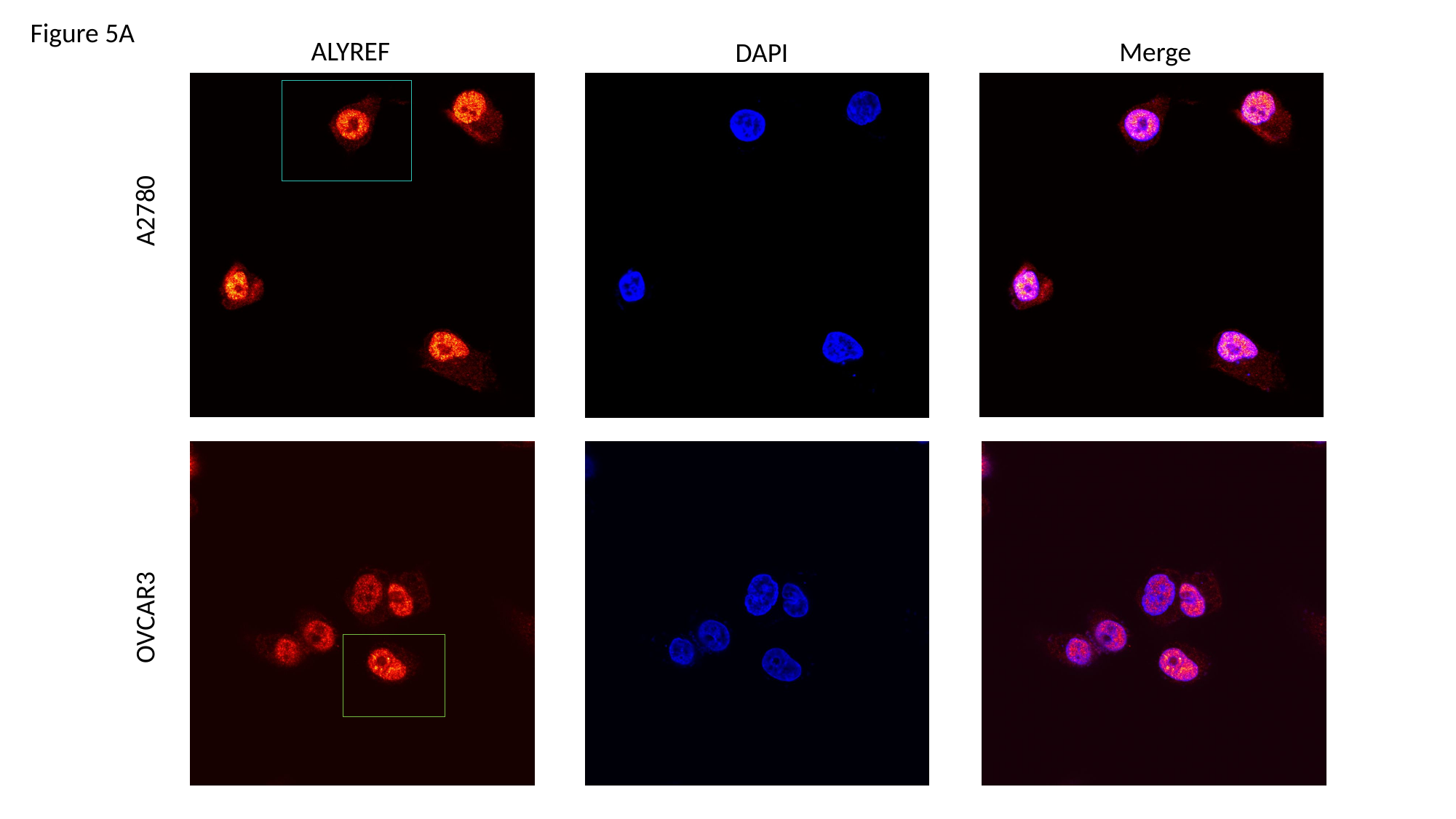

Figure 5A
ALYREF
Merge
DAPI
A2780
OVCAR3

Supplement: Supplementary file 15 — Source data Fig. 5 [file 44318_2025_657_MOESM15_ESM.zip › Source Data for Figure 5/Source Data for Figure 5A.pptx]

## Slide 1
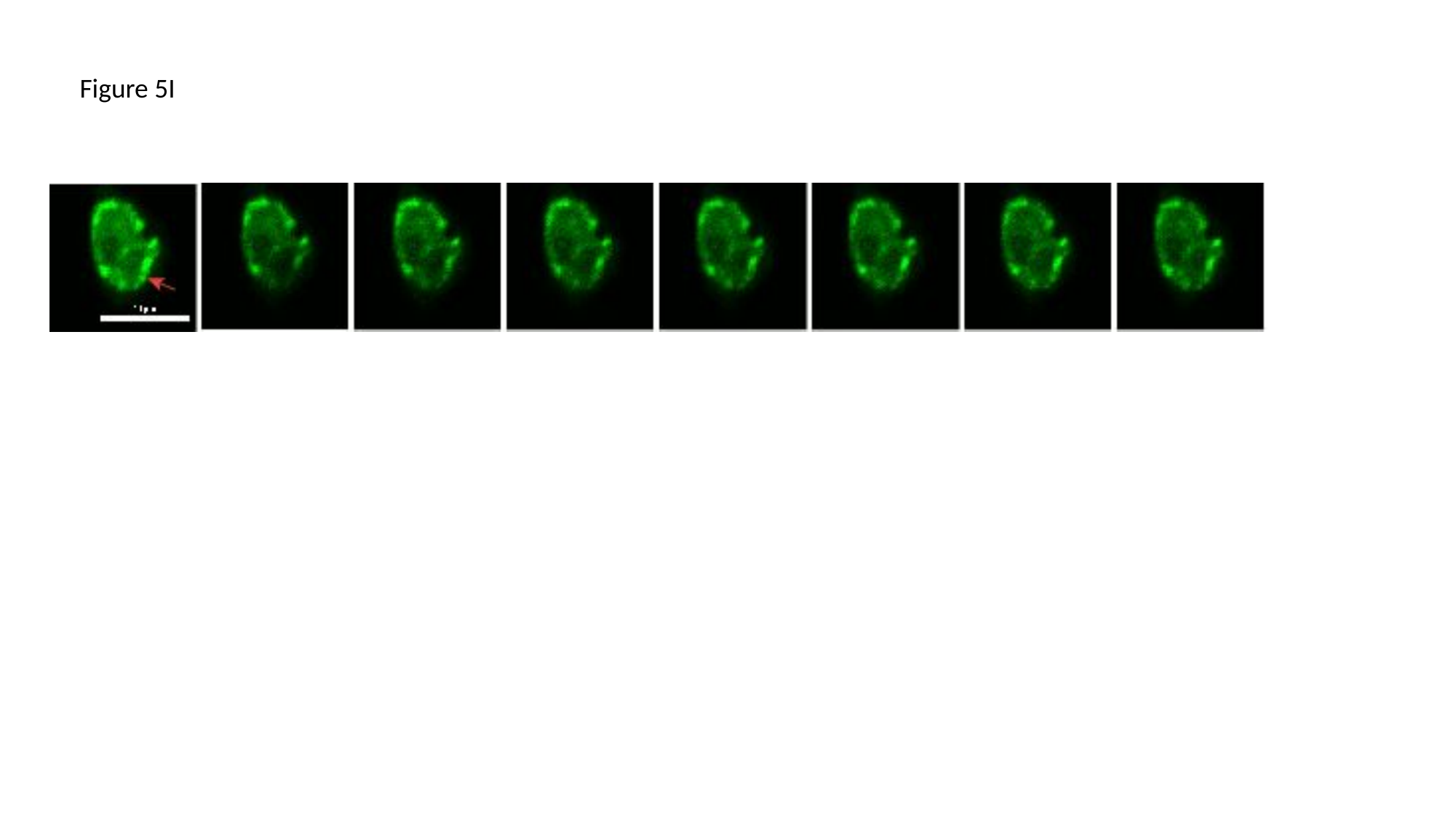

Figure 5I

Supplement: Supplementary file 15 — Source data Fig. 5 [file 44318_2025_657_MOESM15_ESM.zip › Source Data for Figure 5/Source Date for Figure 5I.pptx]

## Slide 1
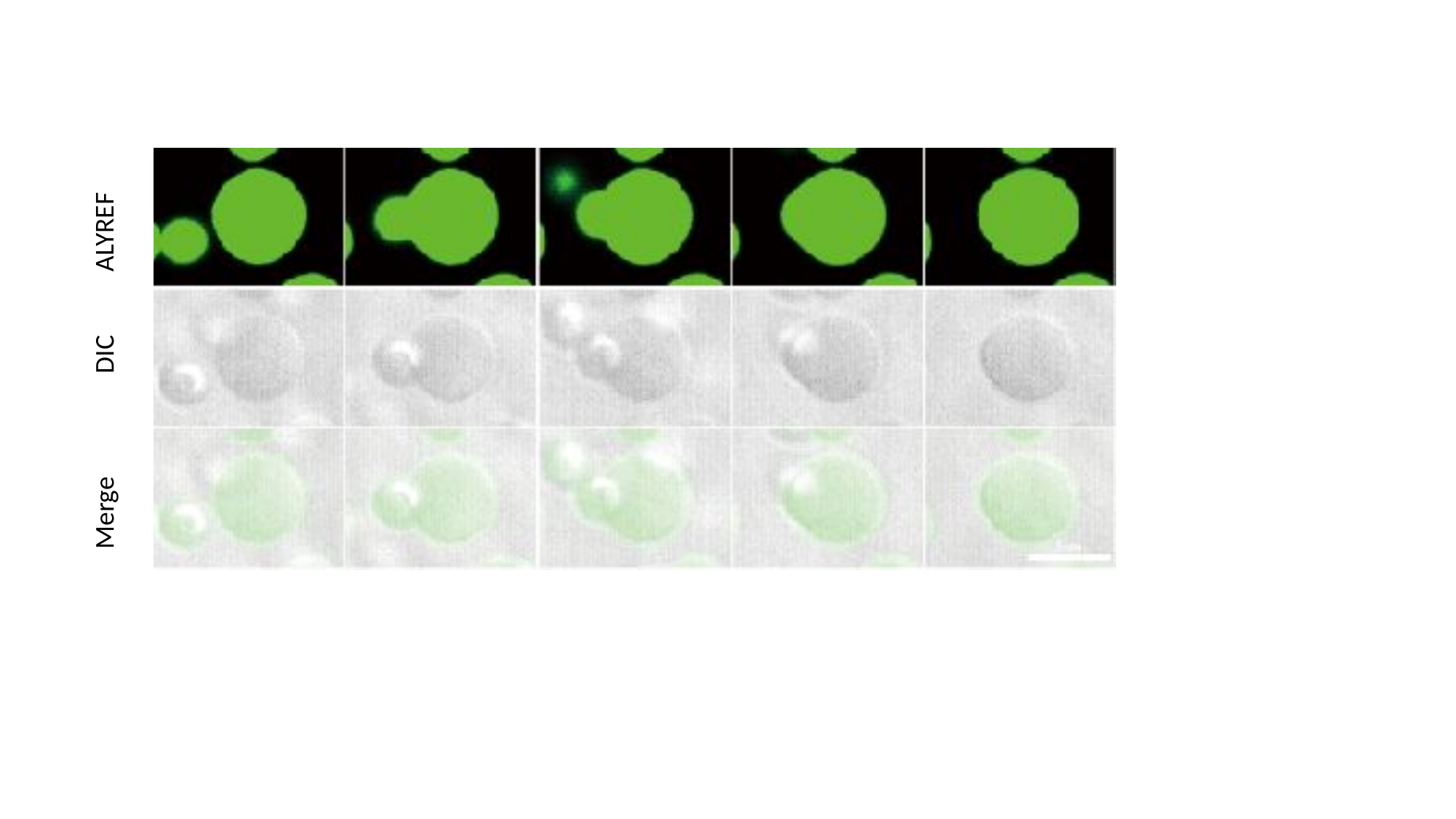

ALYREF
DIC
Merge

Supplement: Supplementary file 15 — Source data Fig. 5 [file 44318_2025_657_MOESM15_ESM.zip › Source Data for Figure 5/Source Date for Figure 5D.pptx]

## Slide 1
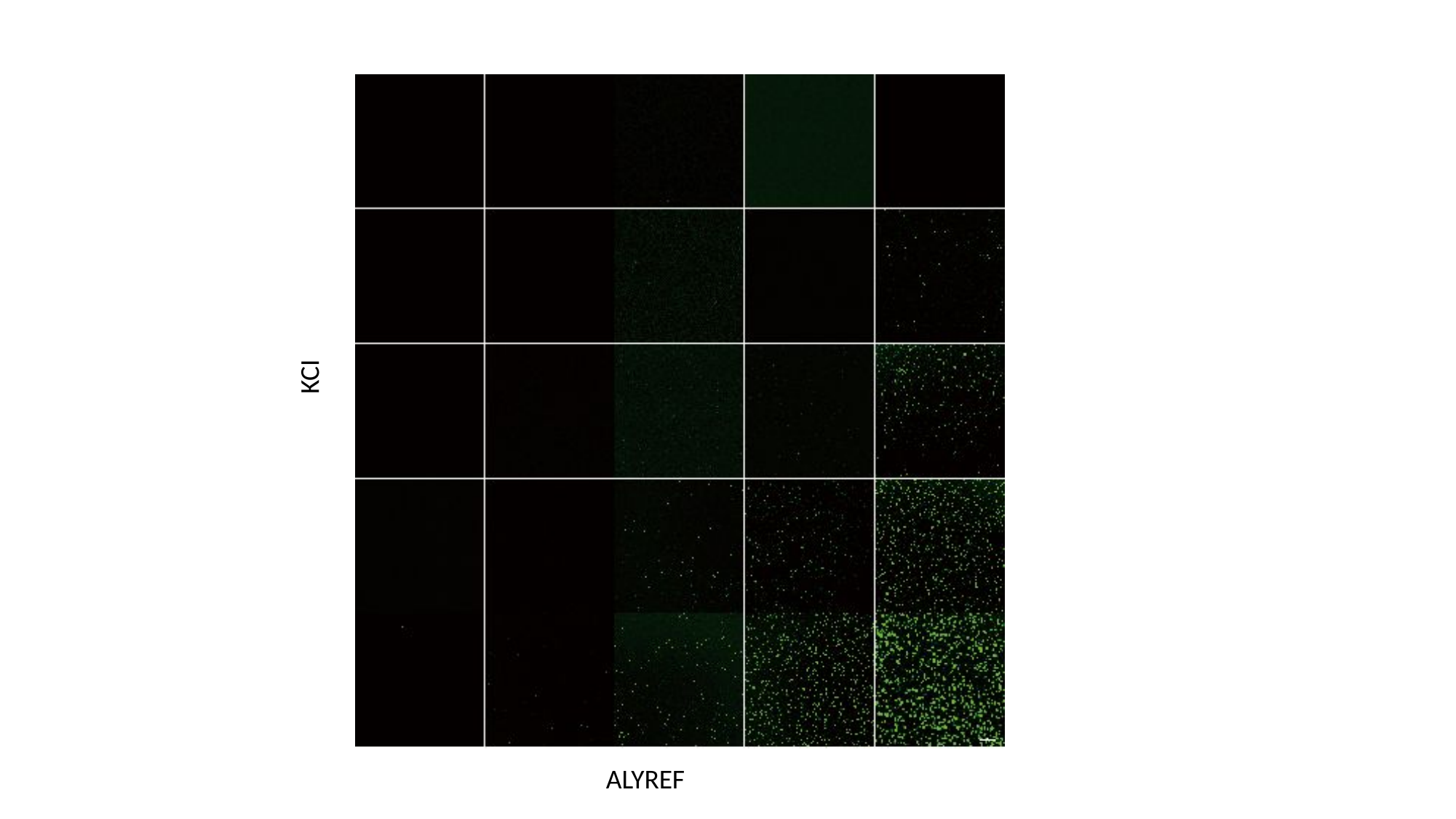

KCI
ALYREF

Supplement: Supplementary file 15 — Source data Fig. 5 [file 44318_2025_657_MOESM15_ESM.zip › Source Data for Figure 5/Source Date for Figure 5E.pptx]

## Slide 1
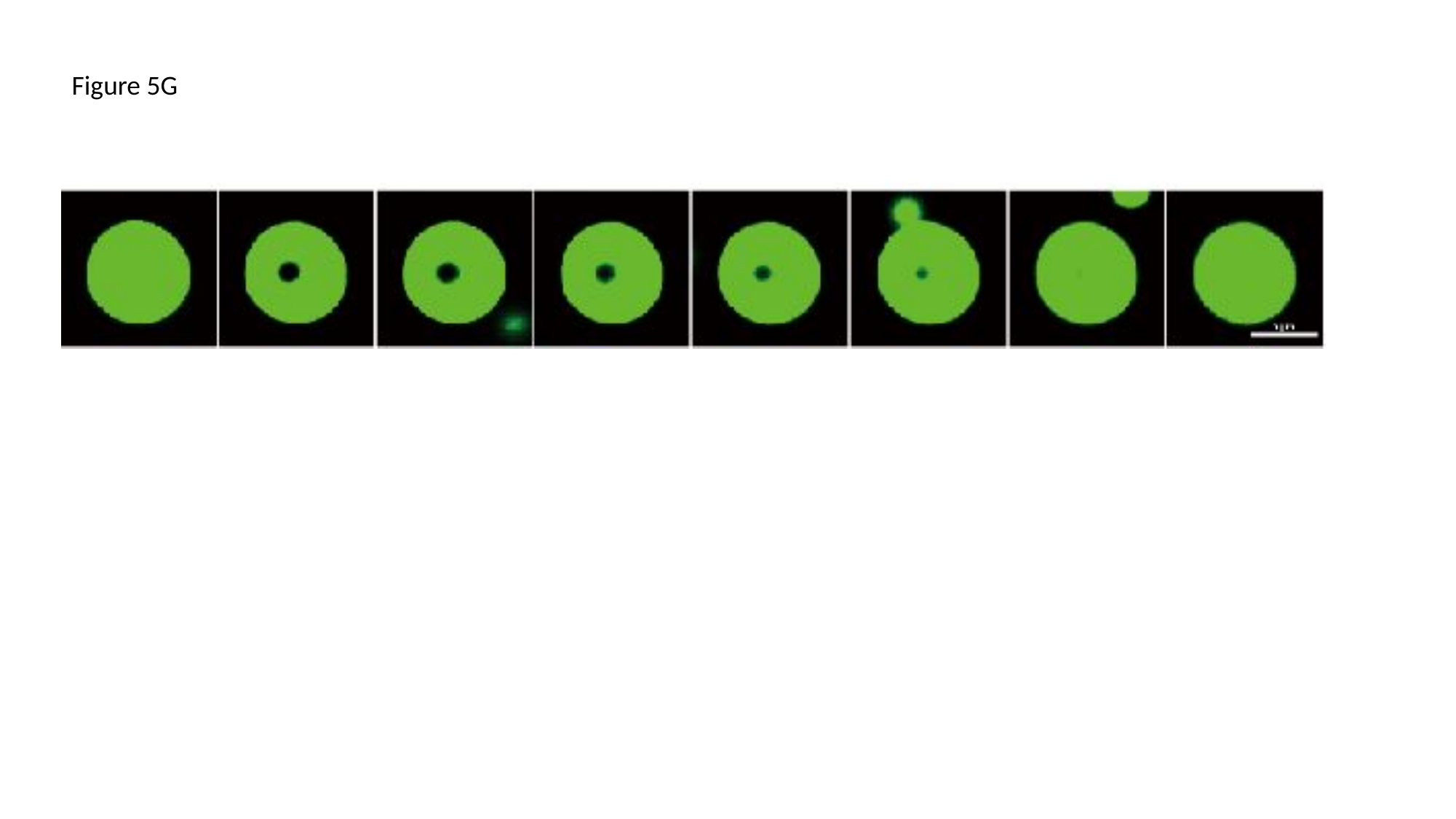

Figure 5G

Supplement: Supplementary file 15 — Source data Fig. 5 [file 44318_2025_657_MOESM15_ESM.zip › Source Data for Figure 5/Source Date for Figure 5G.pptx]

## Slide 1
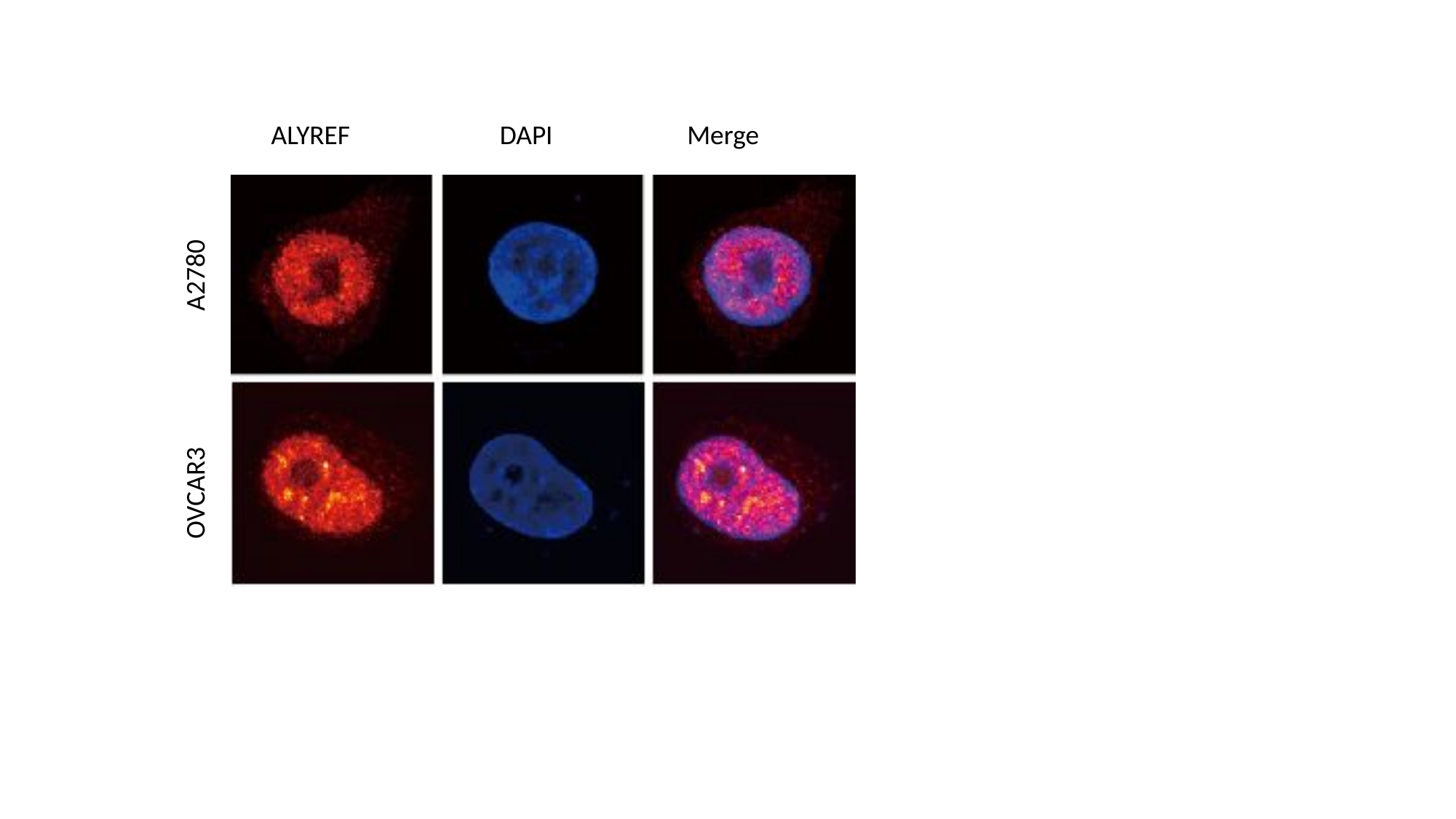

ALYREF
DAPI
Merge
A2780
OVCAR3

Supplement: Supplementary file 15 — Source data Fig. 5 [file 44318_2025_657_MOESM15_ESM.zip › Source Data for Figure 5/Source Date for Figure 5A.pptx]

## Slide 1
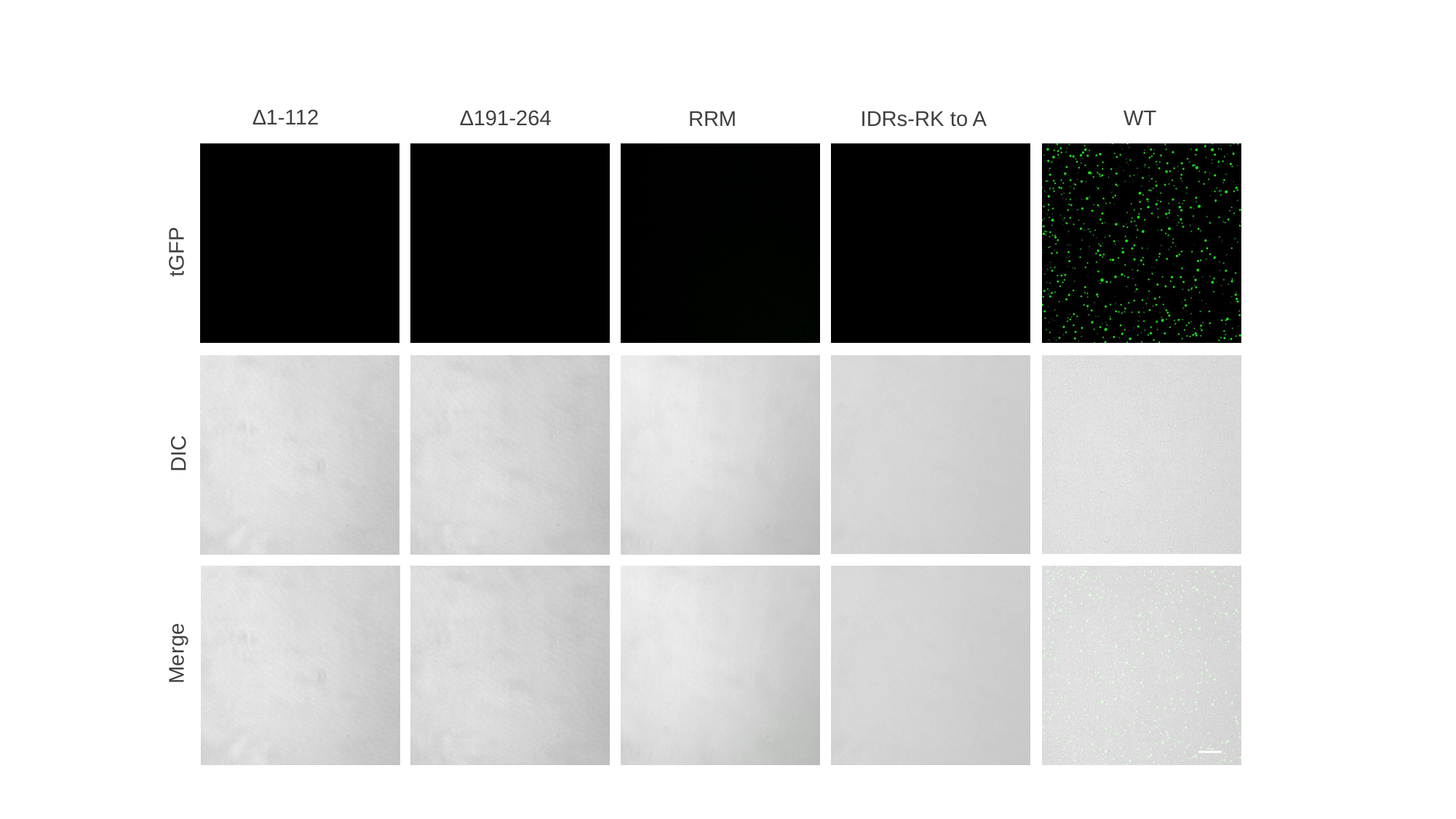

∆1-112
∆191-264
WT
RRM
IDRs-RK to A
tGFP
DIC
Merge

Supplement: Supplementary file 16 — Source data Fig. 6 [file 44318_2025_657_MOESM16_ESM.zip › Source Data for Figure 6/Source Data for Figure 6B.pptx]

## Slide 1
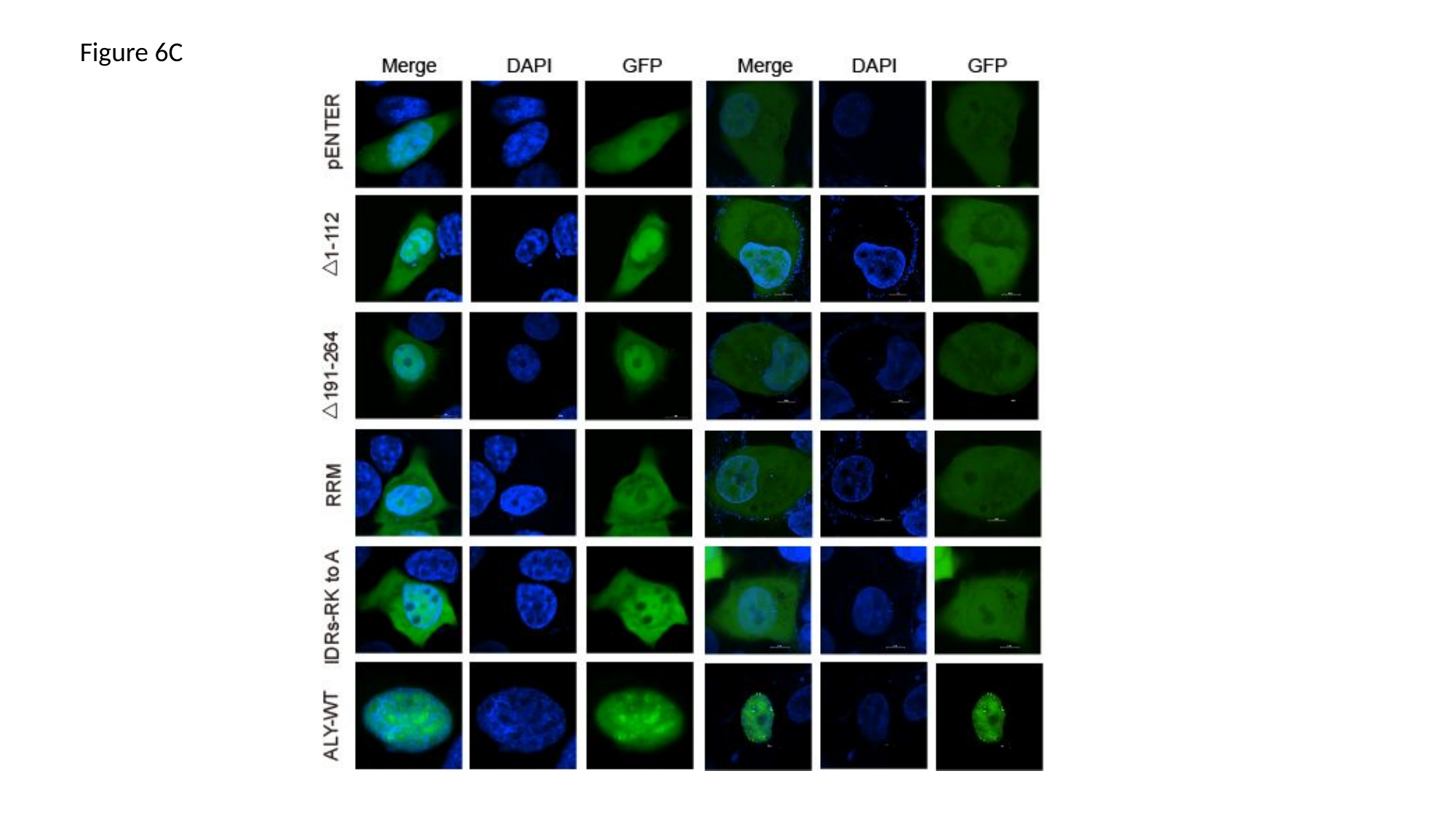

Figure 6C

Supplement: Supplementary file 16 — Source data Fig. 6 [file 44318_2025_657_MOESM16_ESM.zip › Source Data for Figure 6/Source Date for Figure 6C.pptx]

## Slide 1
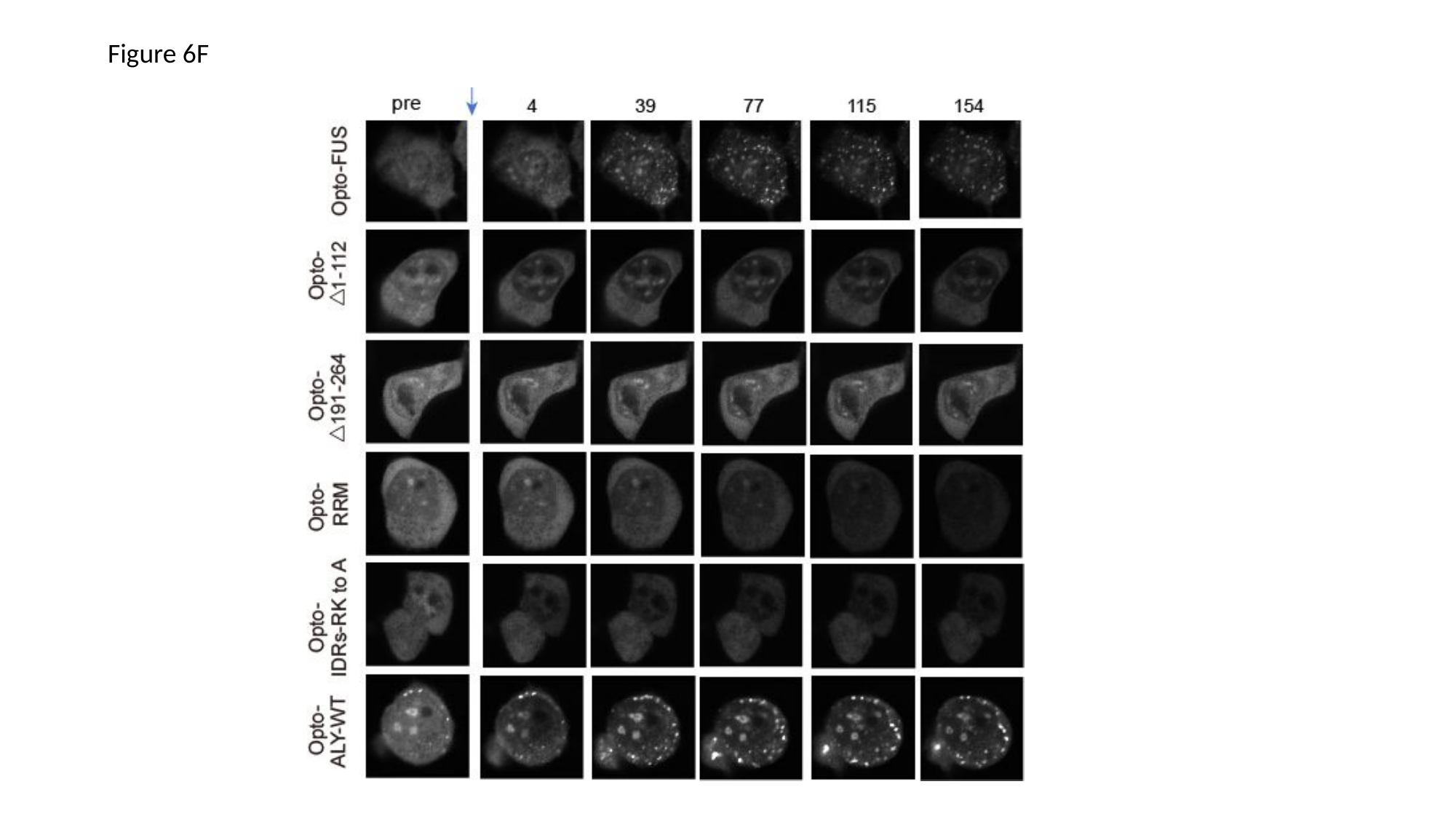

Figure 6F

Supplement: Supplementary file 16 — Source data Fig. 6 [file 44318_2025_657_MOESM16_ESM.zip › Source Data for Figure 6/Source Date for Figure 6F.pptx]

## Slide 1
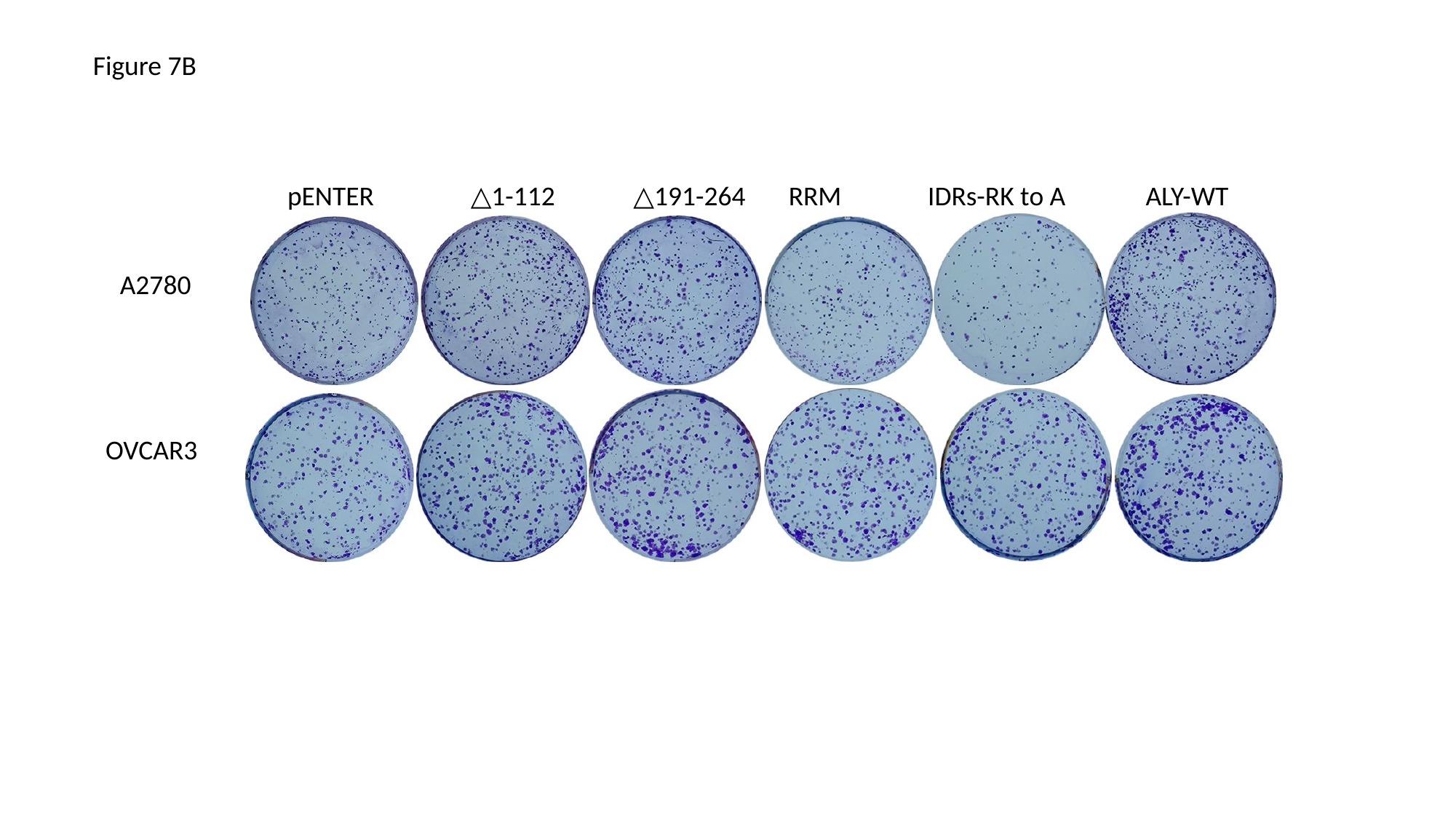

Figure 7B
pENTER	 △1-112	 △191-264 RRM IDRs-RK to A ALY-WT
A2780
OVCAR3

Supplement: Supplementary file 17 — Source data Fig. 7 [file 44318_2025_657_MOESM17_ESM.zip › Source Data for Figure 7/Source Date for Figure 7B.pptx]

## Slide 1
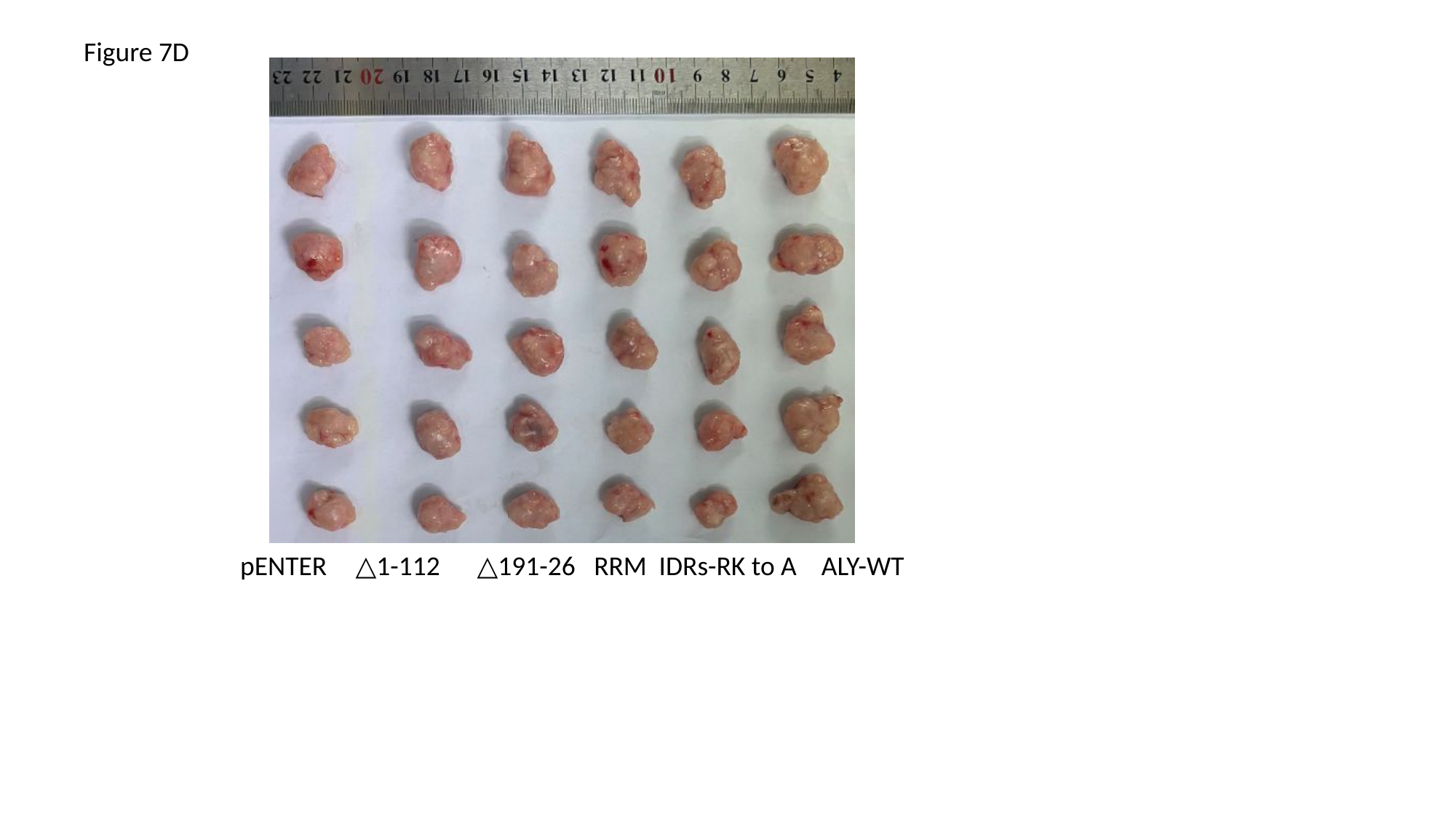

Figure 7D
pENTER	 △1-112	 △191-26 RRM IDRs-RK to A ALY-WT

Supplement: Supplementary file 17 — Source data Fig. 7 [file 44318_2025_657_MOESM17_ESM.zip › Source Data for Figure 7/Source Date for Figure 7D.pptx]

## Slide 1
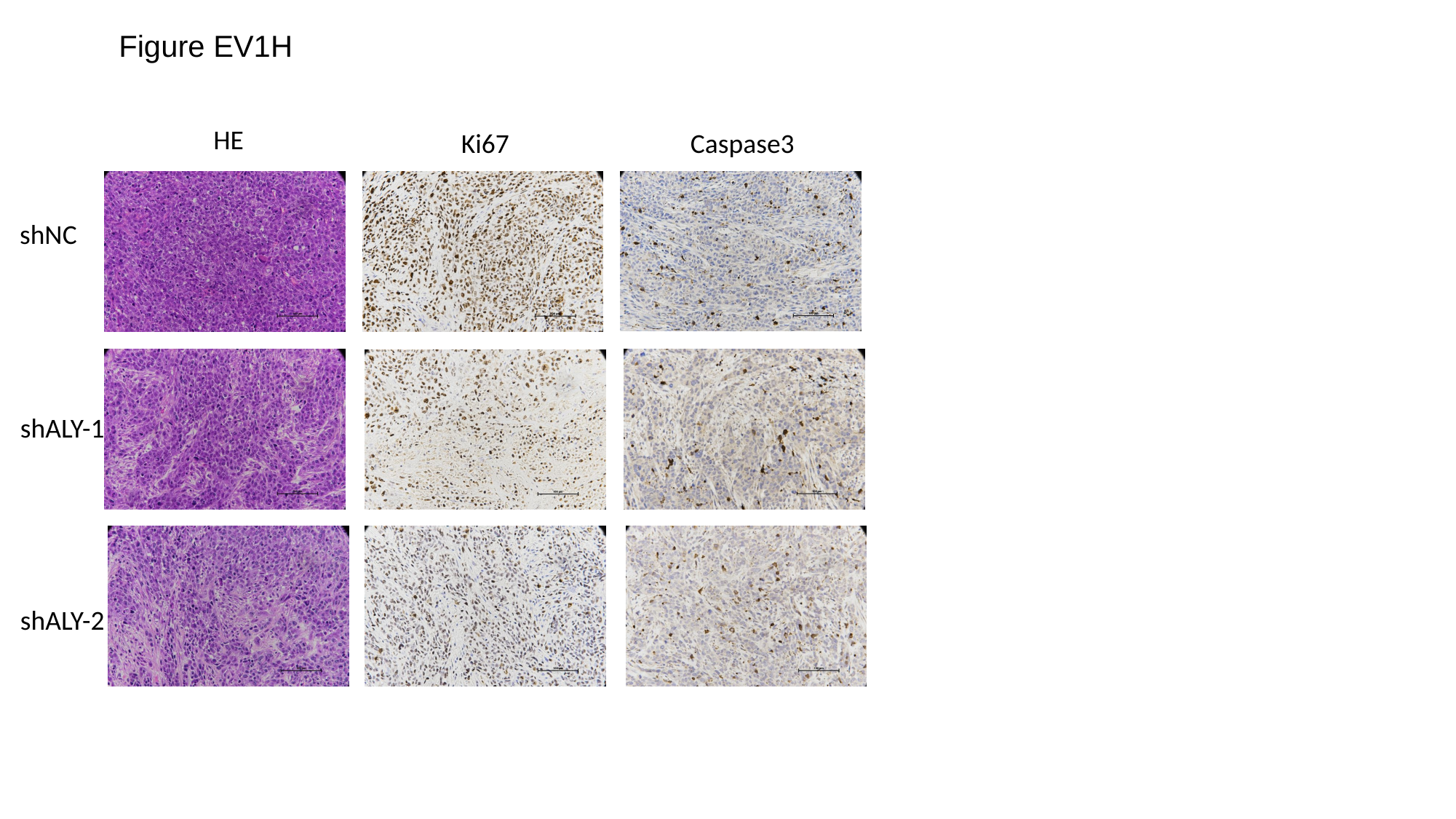

Figure EV1H
HE
Ki67
Caspase3
shNC
shALY-1
shALY-2

Supplement: Supplementary file 19 — Figure EV1 Source Data [file 44318_2025_657_MOESM19_ESM.zip › Source Data for Figure EV1/Source Date for Figure EV1H.pptx]

## Slide 1
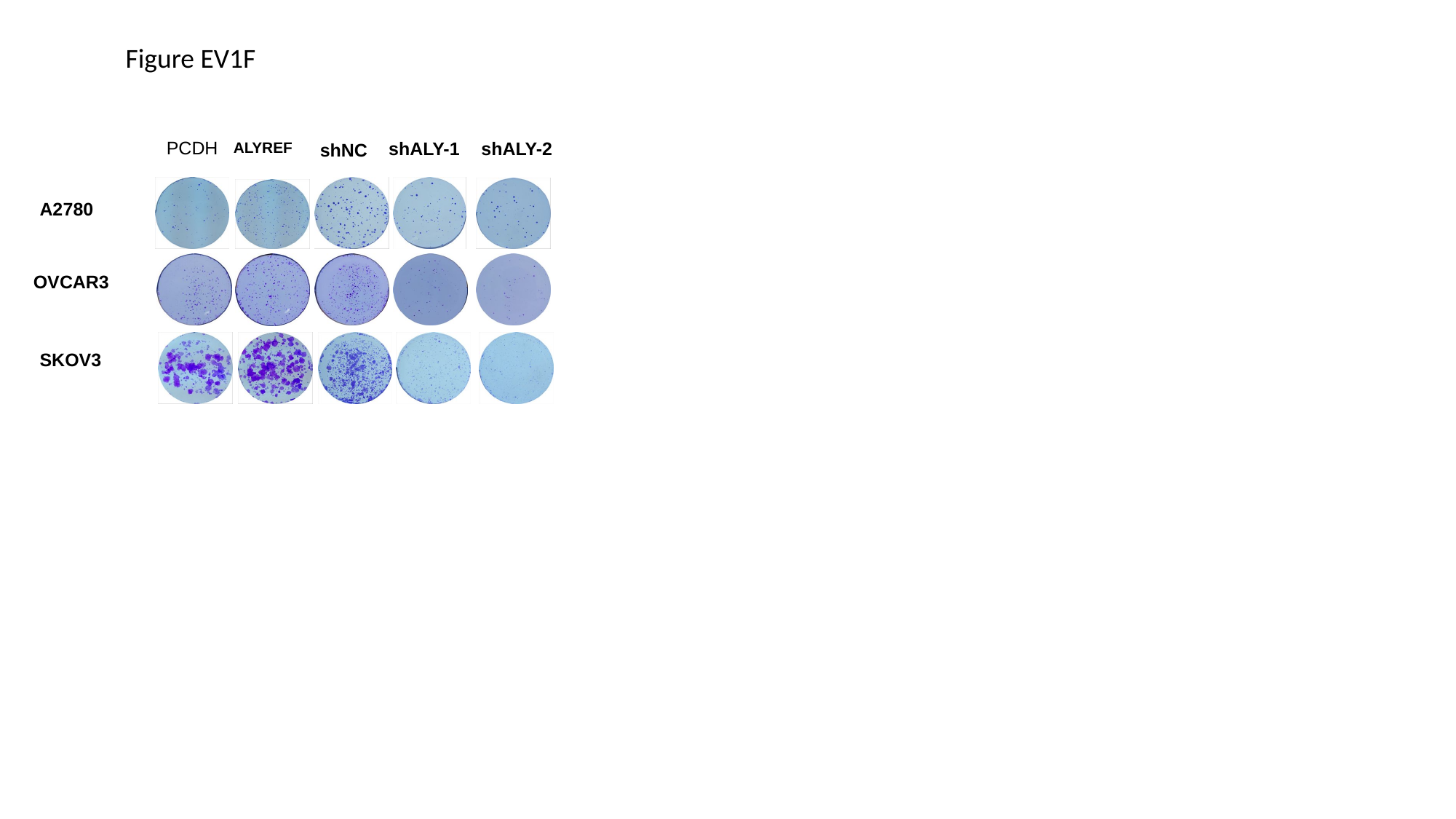

Figure EV1F
PCDH
shALY-1
shALY-2
ALYREF
shNC
A2780
OVCAR3
SKOV3

Supplement: Supplementary file 19 — Figure EV1 Source Data [file 44318_2025_657_MOESM19_ESM.zip › Source Data for Figure EV1/Source Date for Figure EV1F.pptx]

## Slide 1
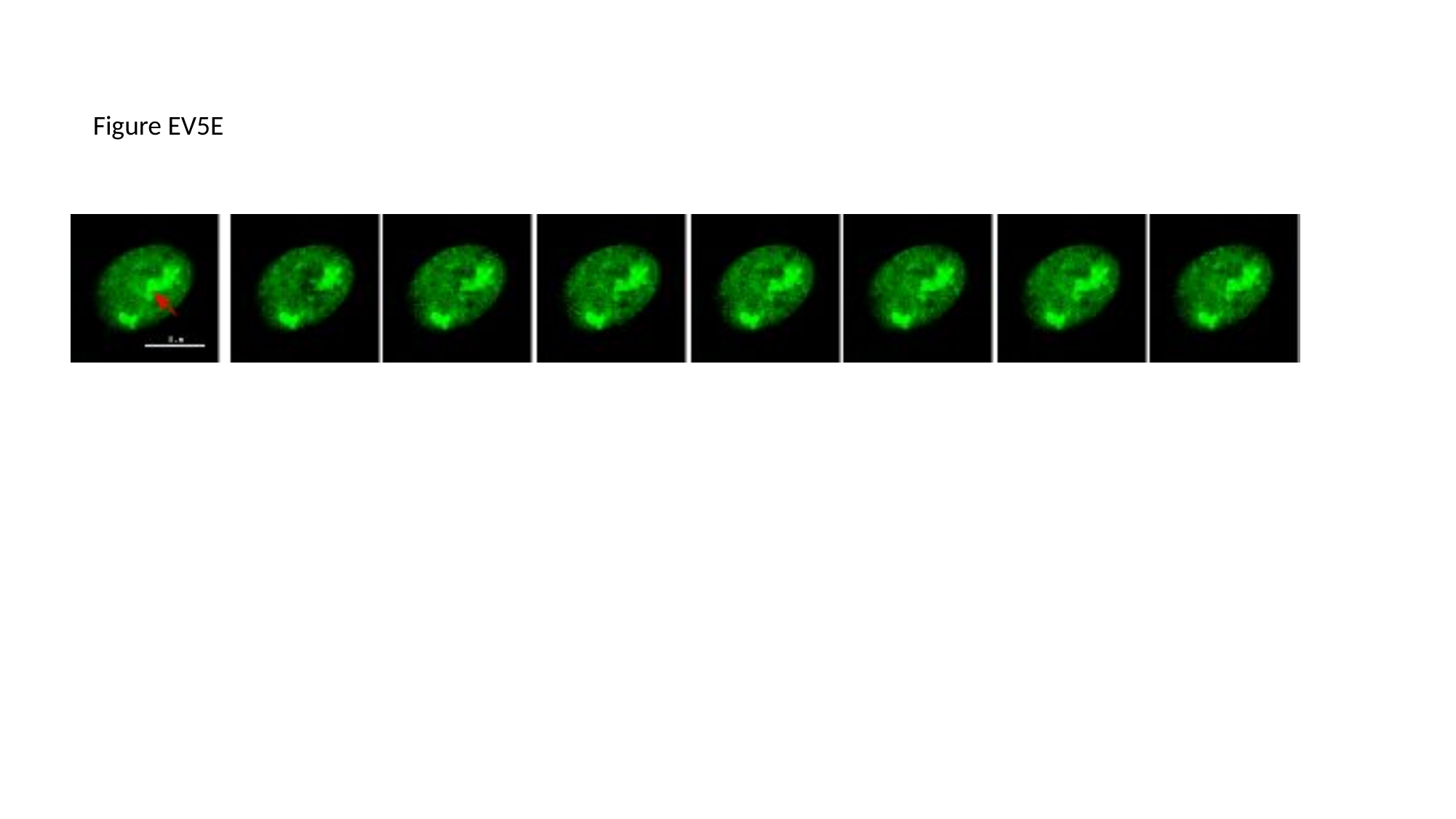

Figure EV5E

Supplement: Supplementary file 23 — Figure EV5 Source Data [file 44318_2025_657_MOESM23_ESM.zip › Source Data for Figure EV5/Source Date for Figure EV5E.pptx]

## Slide 1
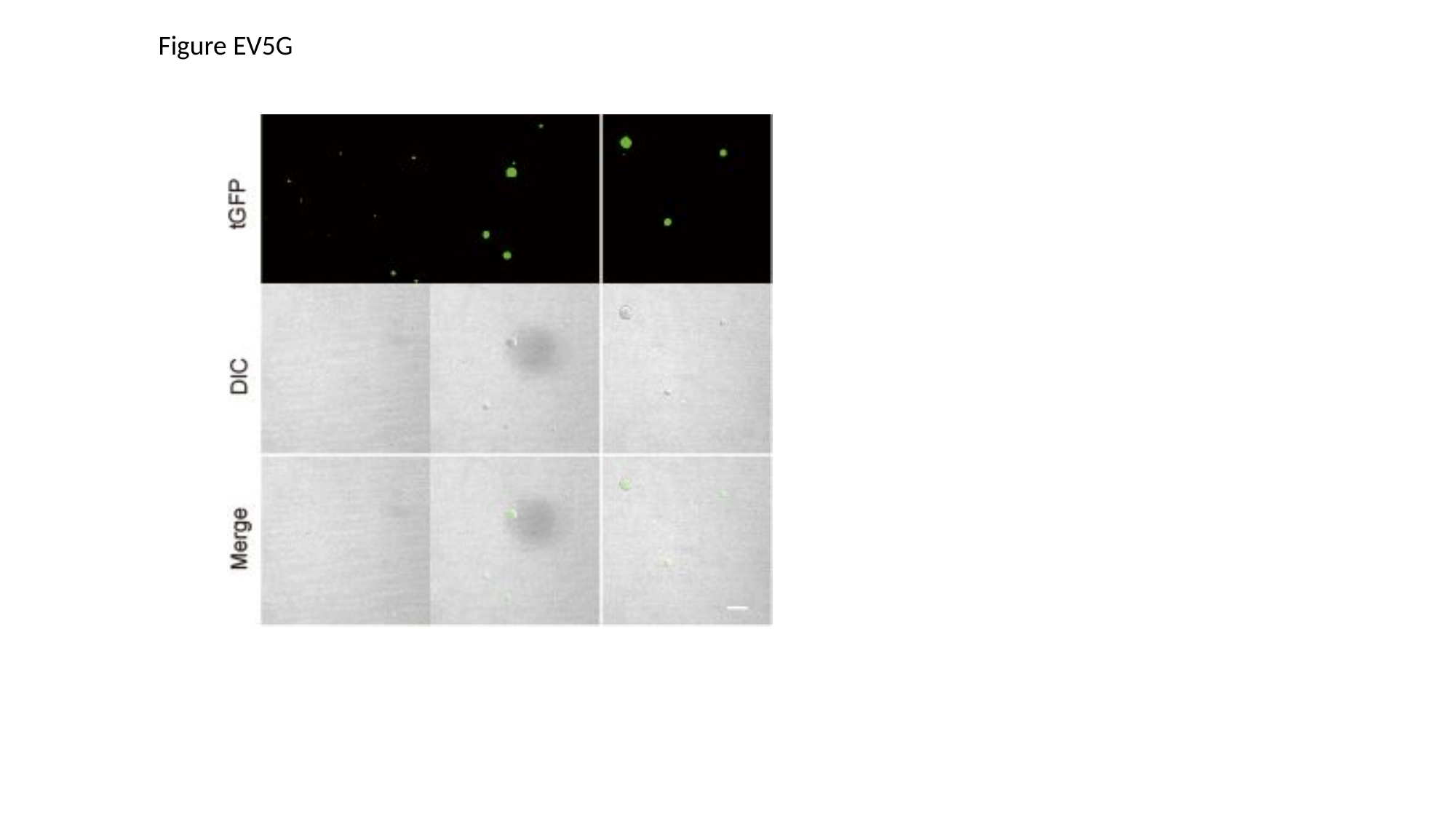

Figure EV5G

Supplement: Supplementary file 23 — Figure EV5 Source Data [file 44318_2025_657_MOESM23_ESM.zip › Source Data for Figure EV5/Source Date for Figure EV5G.pptx]

## Slide 1
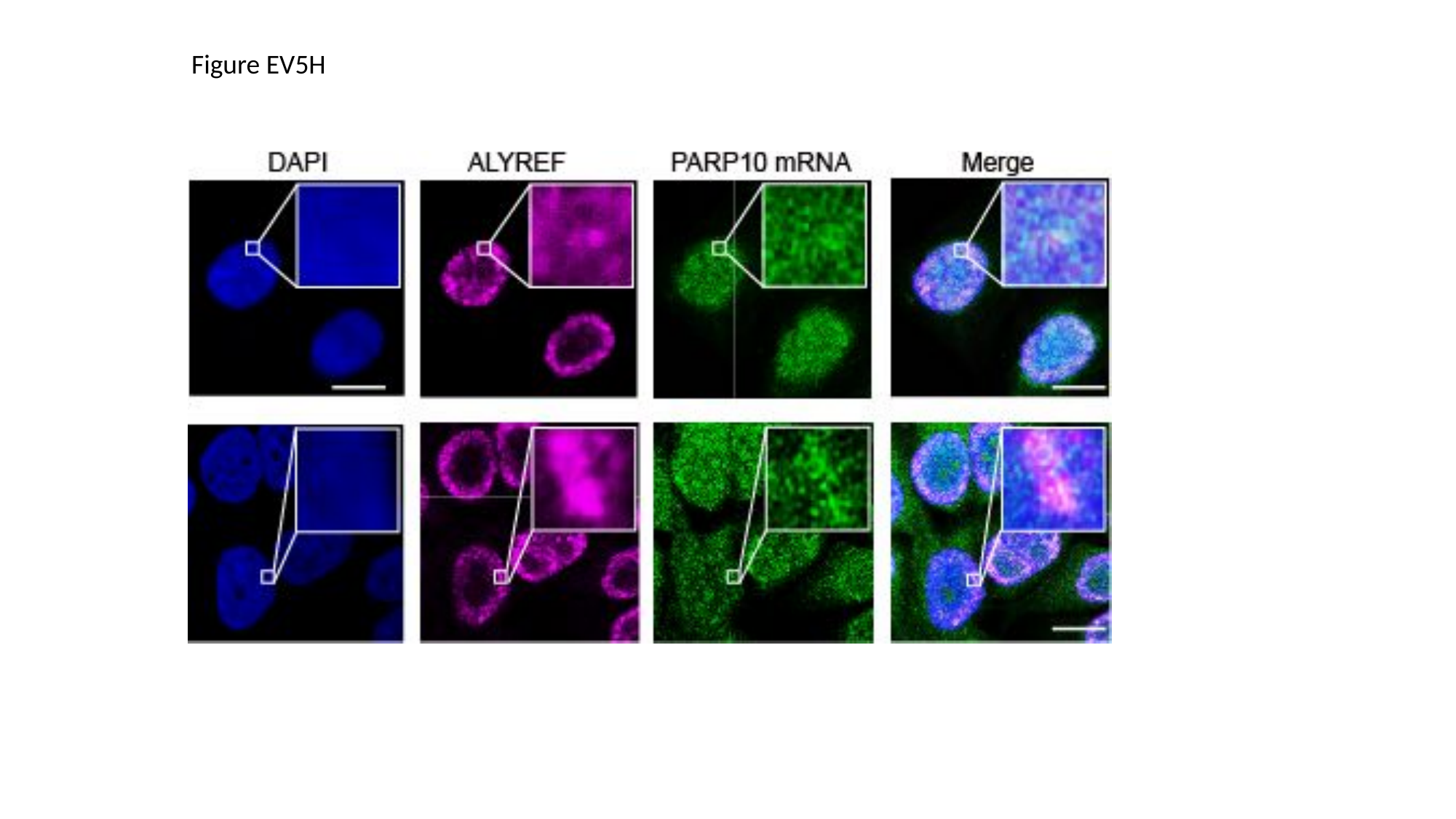

Figure EV5H

Supplement: Supplementary file 23 — Figure EV5 Source Data [file 44318_2025_657_MOESM23_ESM.zip › Source Data for Figure EV5/Source Date for Figure EV5H.pptx]

## Slide 1
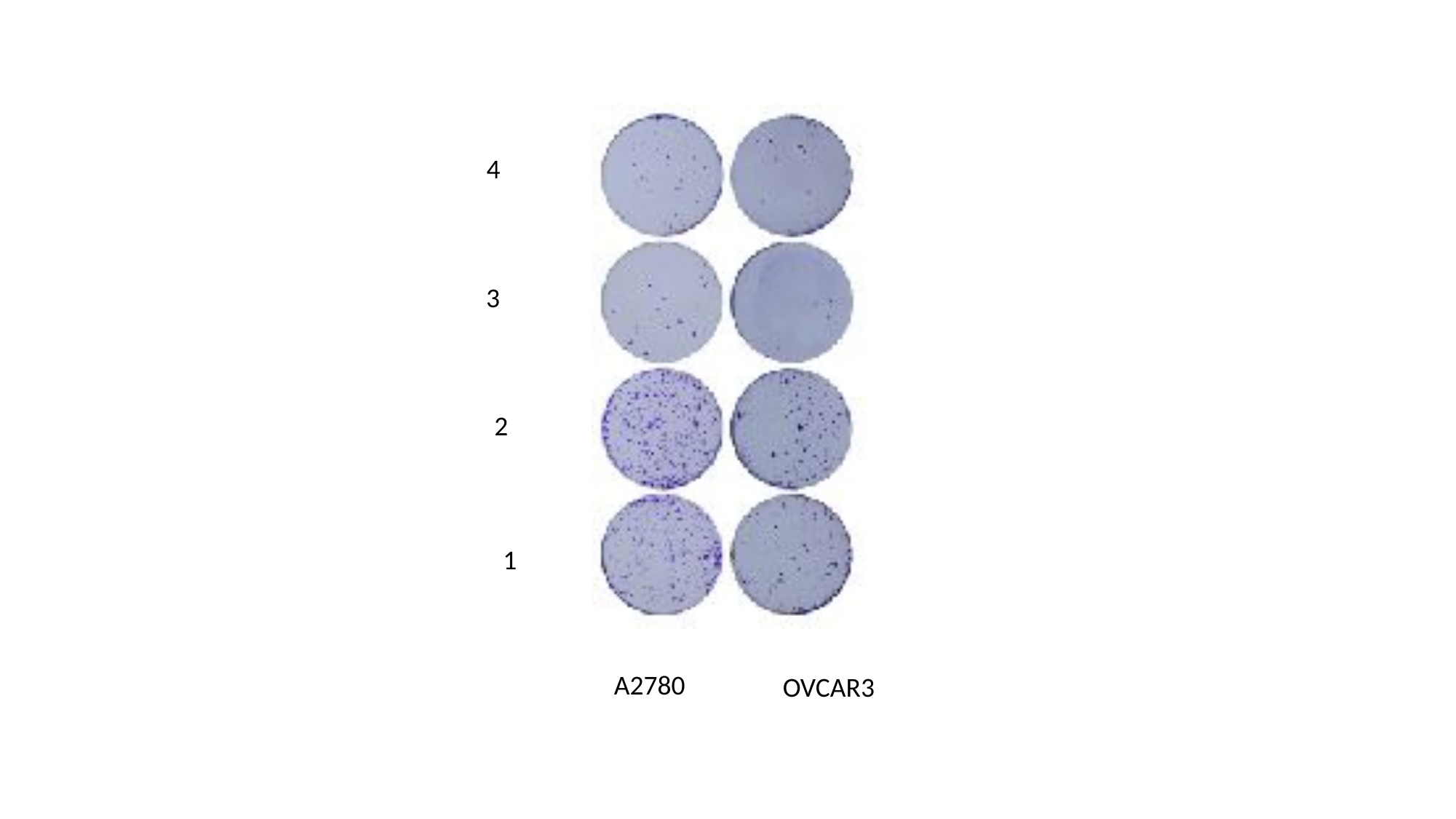

4
3
2
1
A2780
OVCAR3

## Slide 2
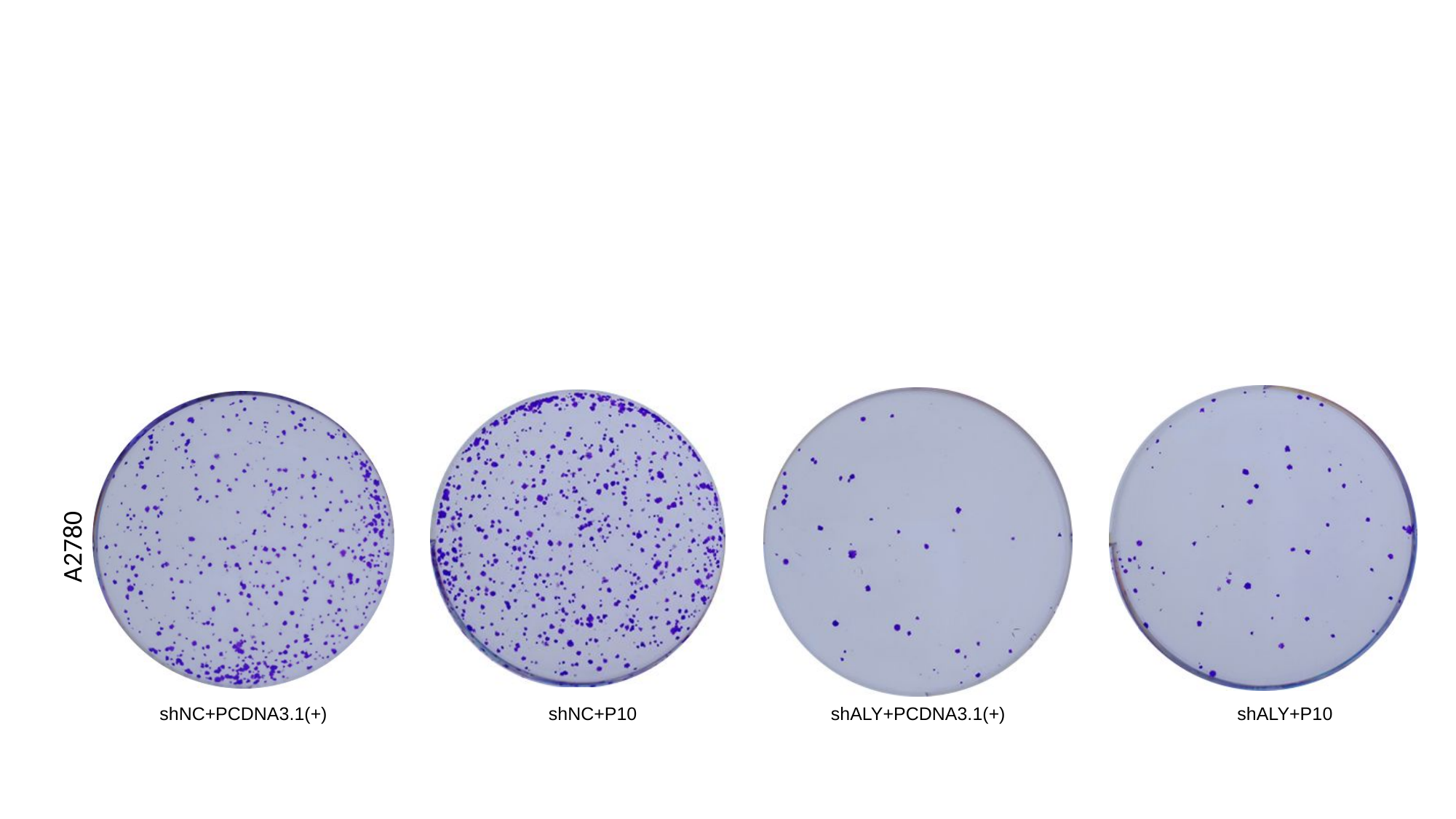

A2780
shNC+PCDNA3.1(+)
shNC+P10
shALY+PCDNA3.1(+)
shALY+P10

Supplement: Supplementary file 25 — Figure EV7 Source Data [file 44318_2025_657_MOESM25_ESM.zip › Source Data for Figure EV7/Source Data for Figure EV7C.pptx]

## Slide 1
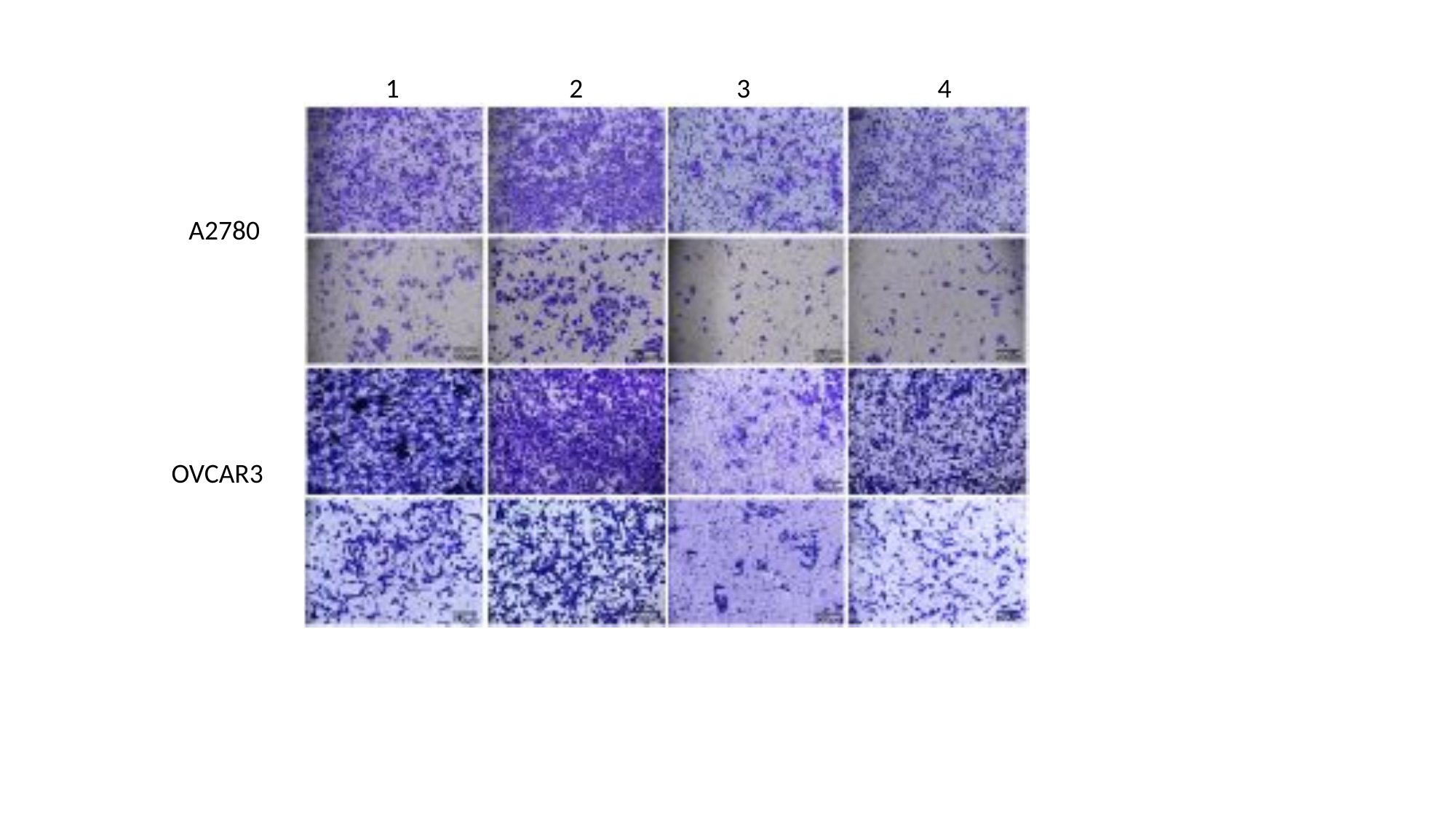

1
2
3
4
A2780
OVCAR3

## Slide 2
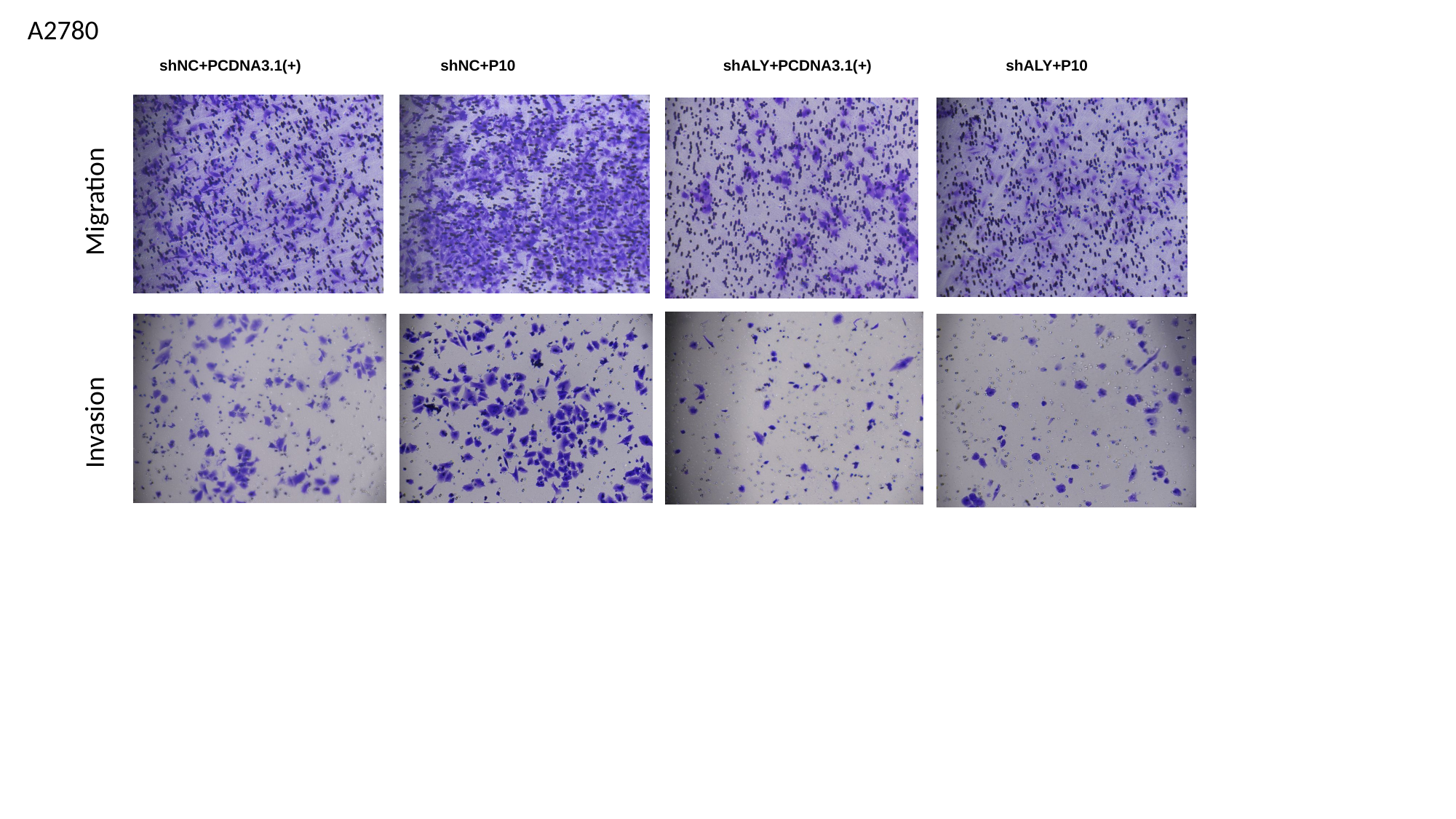

A2780
shNC+PCDNA3.1(+)
shNC+P10
shALY+PCDNA3.1(+)
shALY+P10
Migration
Invasion

Supplement: Supplementary file 25 — Figure EV7 Source Data [file 44318_2025_657_MOESM25_ESM.zip › Source Data for Figure EV7/Source Data for Figure EV7D.pptx]

## Slide 1
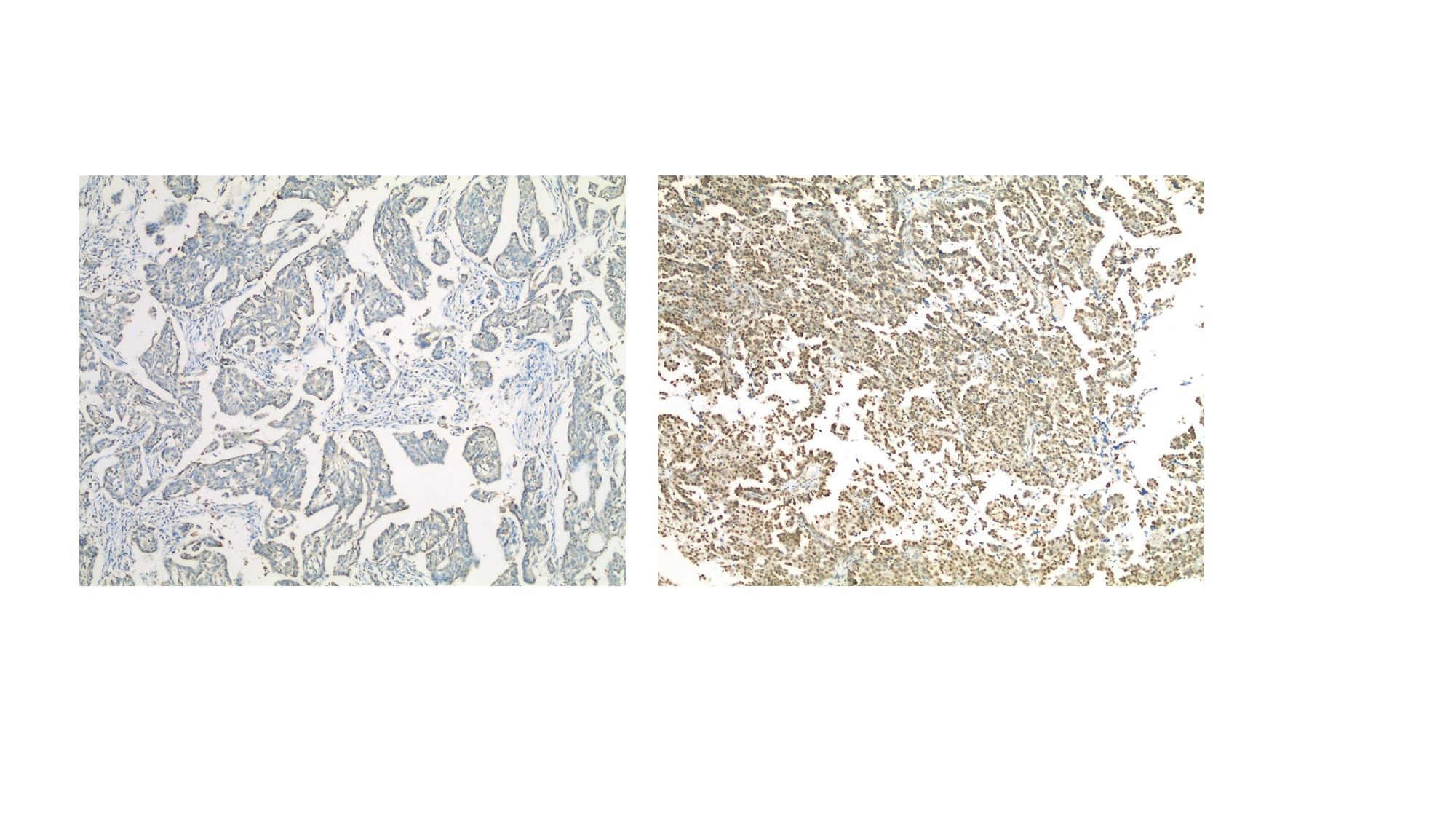

Supplement: Supplementary file 26 — Figure EV8 Source Data [file 44318_2025_657_MOESM26_ESM.zip › Source Data for Figure EV8/Source Data for Figure EV8G.pptx]
