# Supplementary material for: ALYREF condensation stabilizes m5C-modified PARP10 mRNA and promotes PI3K-AKT signaling in ovarian cancer
Source: EMBO J. 2025 Dec 1;45(2):471–503. doi: 10.1038/s44318-025-00657-0 (PMC12811383; doi:10.1038/s44318-025-00657-0)
Supplement: Supplementary file 12 — Source data Fig. 2 [file 44318_2025_657_MOESM12_ESM.zip › Source Data for Figure 2/Source Date for Figure 2F.pptx]

## Slide 1
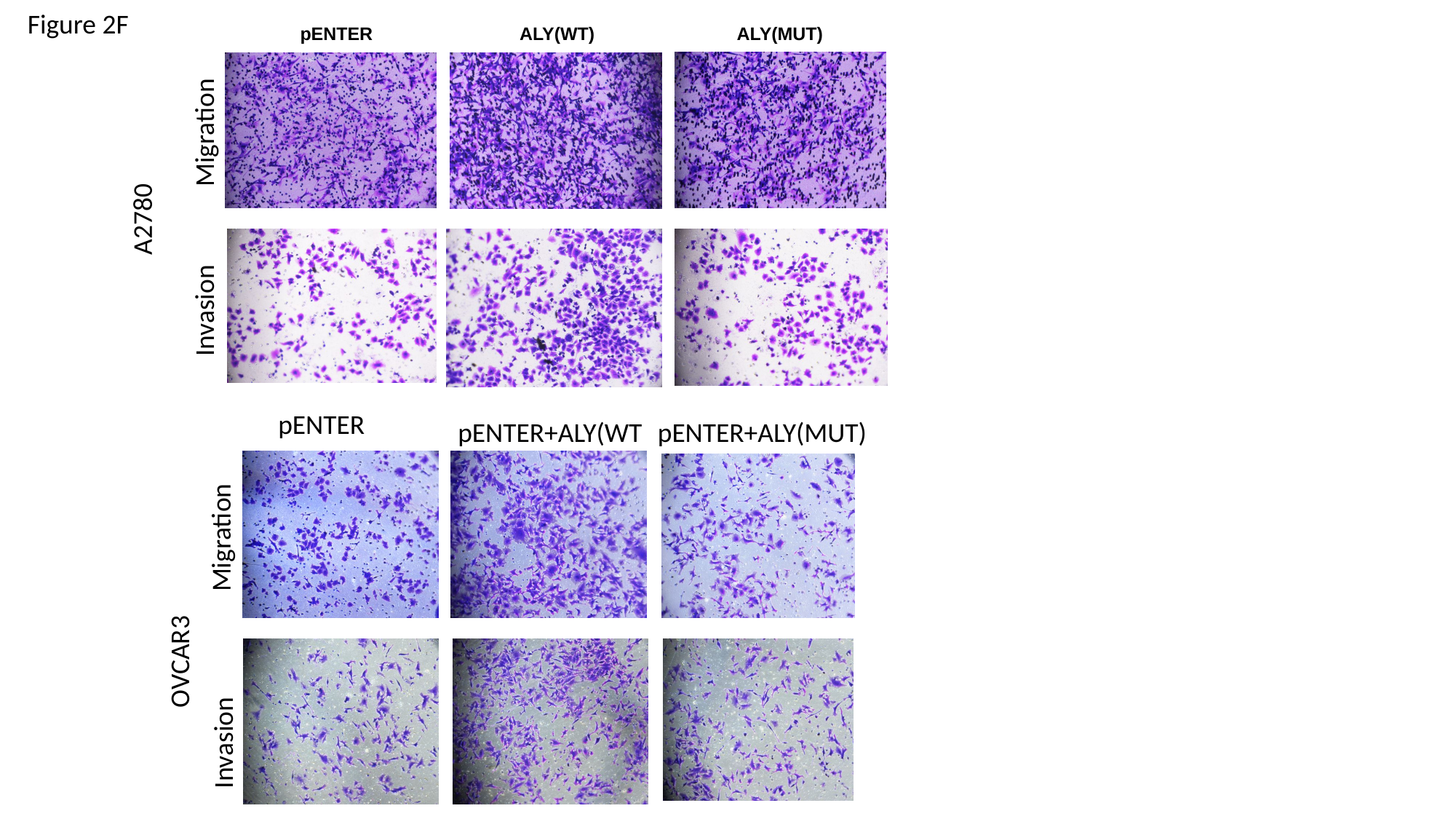

Figure 2F
pENTER
ALY(WT)
ALY(MUT)
Migration
A2780
Invasion
pENTER
Migration
Invasion
pENTER+ALY(WT)
pENTER+ALY(MUT)
OVCAR3
