# Supplementary material for: ALYREF condensation stabilizes m5C-modified PARP10 mRNA and promotes PI3K-AKT signaling in ovarian cancer
Source: EMBO J. 2025 Dec 1;45(2):471–503. doi: 10.1038/s44318-025-00657-0 (PMC12811383; doi:10.1038/s44318-025-00657-0)
Supplement: Supplementary file 19 — Figure EV1 Source Data [file 44318_2025_657_MOESM19_ESM.zip › Source Data for Figure EV1/Source Date for Figure EV1G.pptx]

## Slide 1
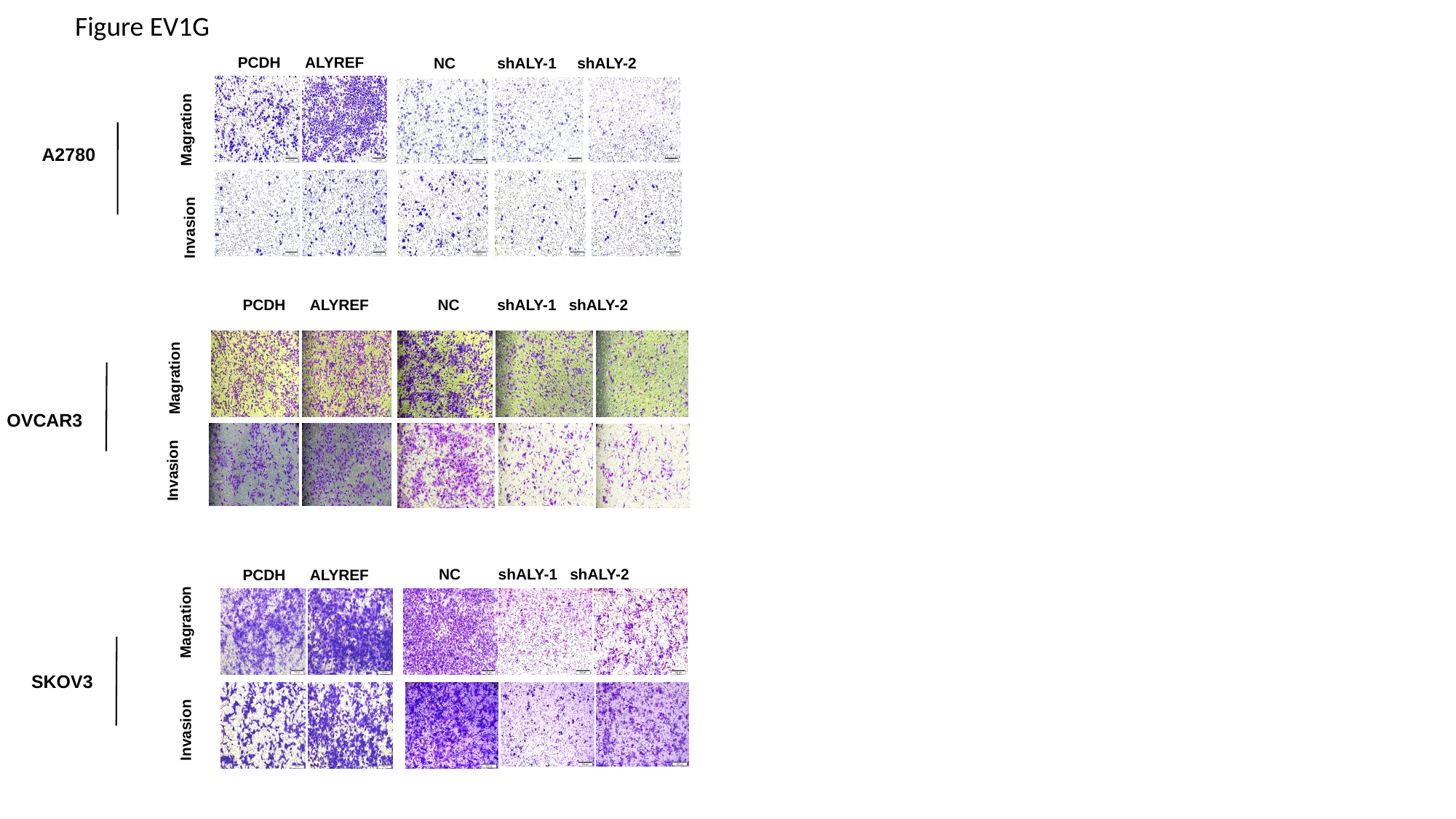

Figure EV1G
PCDH ALYREF
NC shALY-1 shALY-2
Magration
A2780
Invasion
PCDH ALYREF
 NC shALY-1 shALY-2
Magration
OVCAR3
Invasion
 NC shALY-1 shALY-2
PCDH ALYREF
Magration
Invasion
SKOV3
