# Supplementary material for: ALYREF condensation stabilizes m5C-modified PARP10 mRNA and promotes PI3K-AKT signaling in ovarian cancer
Source: EMBO J. 2025 Dec 1;45(2):471–503. doi: 10.1038/s44318-025-00657-0 (PMC12811383; doi:10.1038/s44318-025-00657-0)
Supplement: Supplementary file 27 — Expanded View Figures [file 44318_2025_657_MOESM27_ESM.pdf]

Expanded View Figures

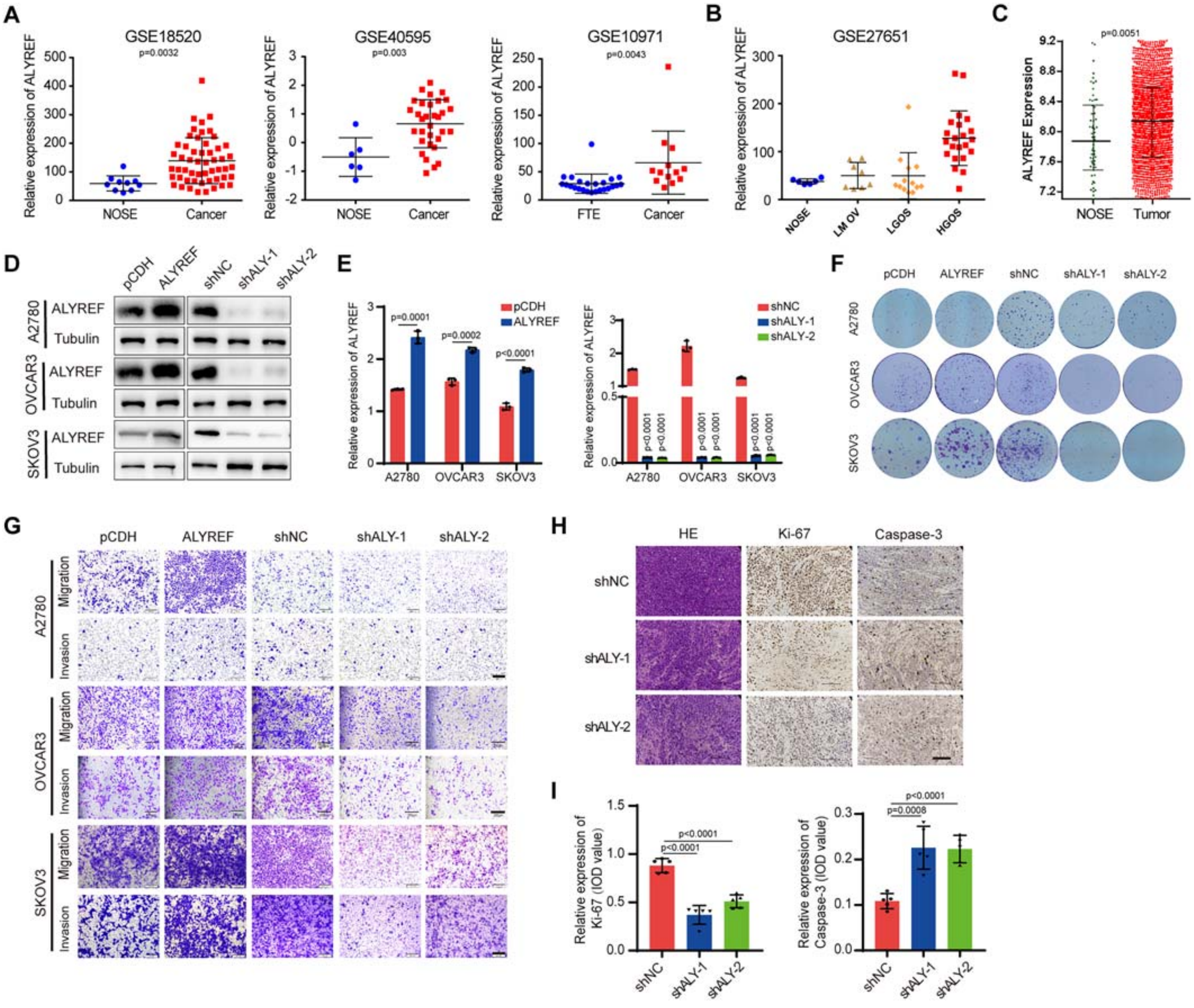

Figure EV1. ALYREF is highly expressed in ovarian cancer and promotes tumorigenesis.

(**A**) Analysis of ALYREF expression in ovarian cancer according to GEO datasets. GSE18520: NOSE ($n = 10$), Cancer ($n = 53$). GSE40595: NOSE ($n = 6$), Cancer ($n = 56$). GSE40595: NOSE ($n = 24$), Cancer ($n = 13$). (**B**) ALYREF was increased in high-grade serous ovarian cancer compared with that in the normal ovarian surface epithelium according to GSE27651. HOSE ($n = 6$),LM OV ($n = 8$), LGOS ($n = 13$), HGOS ($n = 22$). (**C**) ALYREF was overexpressed in ovarian cancer according to the CSIOVDB database. (**D**) Western blotting assays were performed to confirm ALYREF overexpression and knockdown in ovarian cancer cells. (**E**) The expression of the protein was quantified by grayscale in (**D**). $n = 3$ independent experiments. (**F**) The colony formation assays of ovarian cancer cells upon ALYREF overexpression or knockdown. (**G**) The transwell migration and matrigel invasion assays of ovarian cancer cells upon ALYREF overexpression or knockdown. Scale bar, 200 µm. (**H**) The expression of proteins Ki-67 and Caspase-3 was detected in xenografted tumors formed by ovarian cancer cells with ALYREF knockdown through immunohistochemical staining. Scale bar, 100 µm. (**I**) Statistical analysis results of Ki-67 and Caspase-3 positive staining in xenografted tumors ($n = 5$). Data are shown as means ± S.D. *P* value was calculated by one-way ANOVA test with multiple comparisons (**E**, **I**) or unpaired two-sided Student's *t* test (**A**, **C**, **E**). Source data are available online for this figure.

 © The Author(s)

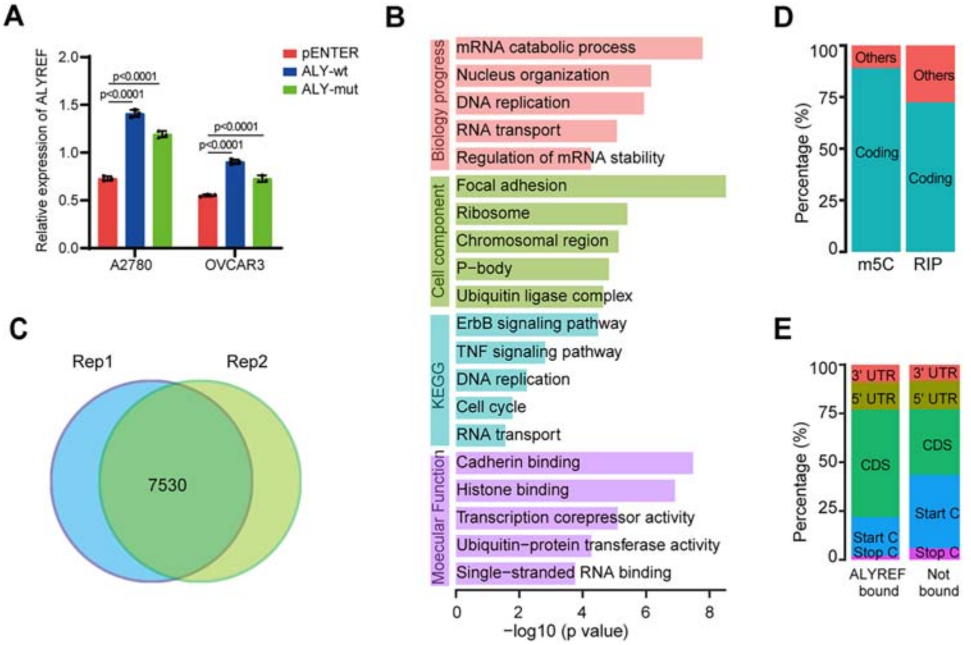

Figure EV2. ALYREF facilitates ovarian cancer progression in an m^{5}C-dependent manner.

(**A**) The expression of the protein was quantified by grayscale in ovarian cancer cells with wild-type or mutated ALYREF overexpression. (**B**) KEGG enrichment analysis revealed that m^{5}C-modified transcripts were enriched in signaling pathways such as ErbB, TNF, and cell cycle. (**C**) RNA-BisSeq analysis showed 7530 transcripts with m^{5}C modification. (**D**) RNA species of ALYREF-binding or m^{5}C-modified transcripts. (**E**) Distribution of m^{5}C modification sites on ALYREF-binding transcripts or not-binding transcripts. $n = 3$ independent experiments (**A**). Data are shown as means ± S.D. *P* value was calculated by one-way ANOVA test with multiple comparisons (**A**). Source data are available online for this figure.

© The Author(s)

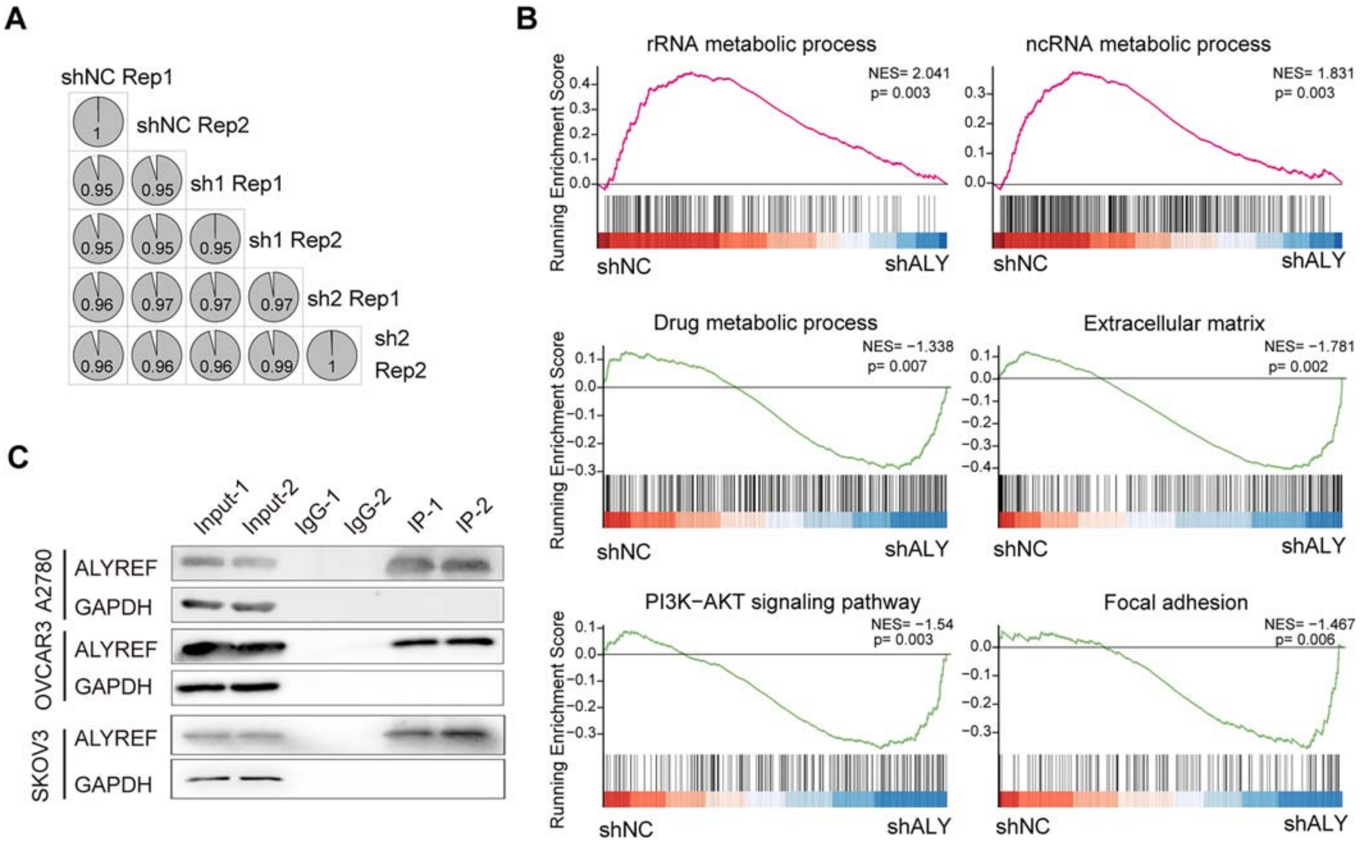

Figure EV3. Identification of ALYREF-regulated transcripts by RNA-seq, RIP-seq, and RNA-BisSeq.

(**A**) Biological repetition analysis of RNA-seq data. (**B**) The GSEA diagram showed that the functions of genes were downregulated upon ALYREF knockdown. (**C**) Western blotting confirmed ALYREF immunoprecipitation in RIP assays. *P* value was calculated by permutation test. Source data are available online for this figure.

 © The Author(s)

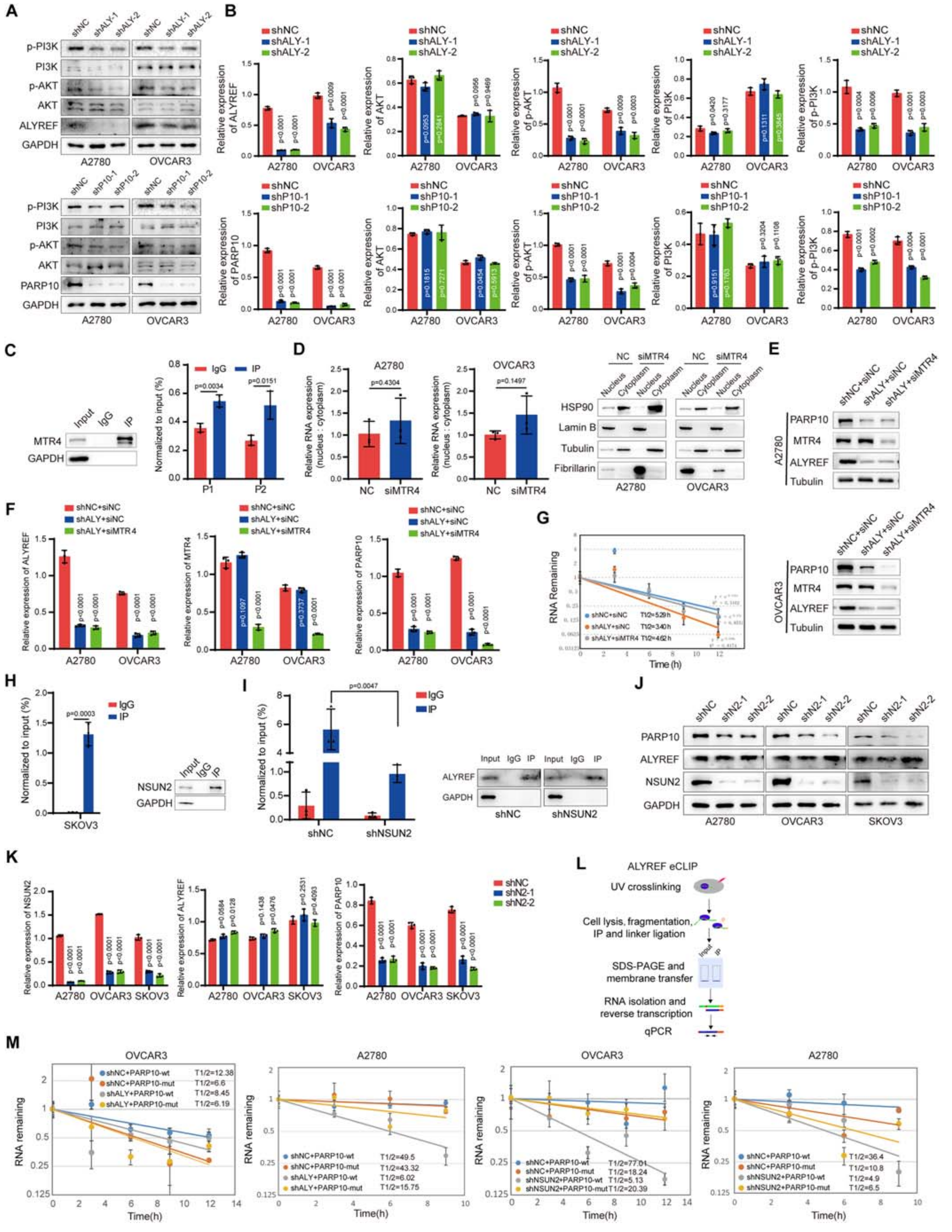
A
shNC
shALY-1
shALY-2
p-PI3K
PI3K
p-AKT
AKT
ALYREF
GAPDH
A2780
OVCAR3
shP10-1
shP10-2
PARP10
B
Relative expression of ALYREF
Relative expression of AKT
Relative expression of p-AKT
Relative expression of PI3K
Relative expression of p-PI3K
Relative expression of PARP10
C
Input
IgG
IP
MTR4
Normalized to input (%)
p=0.0034
p=0.0151
P1
P2
D
Relative RNA expression (nucleus : cytoplasm)
p=0.4304
p=0.1497
NC
siMTR4
HSP90
Lamin B
Tubulin
Fibrillarin
Nucleus
Cytoplasm
E
shNC+siNC
shALY+siNC
shALY+siMTR4
F
Relative expression of ALYREF
Relative expression of MTR4
Relative expression of PARP10
G
RNA Remaining
shNC+siNC T1/2=5.29 h
shALY+siNC T1/2=3.40 h
shALY+siMTR4 T1/2=4.62 h
Time (h)
H
p=0.0003
NSUN2
SKOV3
I
p=0.0047
shNC
shNSUN2
J
shN2-1
shN2-2
K
Relative expression of NSUN2
L
ALYREF eCLIP
UV crosslinking
Cell lysis, fragmentation, IP and linker ligation
SDS-PAGE and membrane transfer
RNA isolation and reverse transcription
qPCR
M
RNA remaining
Time(h)
shNC+PARP10-wt T1/2=12.38
shNC+PARP10-mut T1/2=6.6
shALY+PARP10-wt T1/2=8.45
shALY+PARP10-mut T1/2=6.19
shNC+PARP10-wt T1/2=49.5
shNC+PARP10-mut T1/2=43.32
shALY+PARP10-wt T1/2=6.02
shALY+PARP10-mut T1/2=15.75
shNC+PARP10-wt T1/2=77.01
shNC+PARP10-mut T1/2=18.24
shNSUN2+PARP10-wt T1/2=5.13
shNSUN2+PARP10-mut T1/2=20.39
shNC+PARP10-wt T1/2=36.4
shNC+PARP10-mut T1/2=10.8
shNSUN2+PARP10-wt T1/2=4.9
shNSUN2+PARP10-mut T1/2=6.5

Figure EV4. ALYREF regulates PARP10 expression in an m^{5}C-dependent manner.

(**A**) Detecting the protein levels in ovarian cancer cells upon ALYREF knockdown by western blotting. (**B**) The expression of the protein was quantified by grayscale in (**A**). (**C**) RIP assays detecting the interaction between MTR4 and PARP10 mRNA. (**D**) RT-qPCR detecting the expression level of PARP10 mRNA in nucleocytoplasmic separation assays. (**E**) Effect of ALYREF knockdown on the protein level of MTR4 in A2780 and OVCAR3 cells. (**F**) The expression of the protein was quantified by grayscale in (**E**). (**G**) ALYREF and MTR4 were knocked down in OVCAR3 cells, and PARP10 mRNA stability was detected. (**H**) RT-qPCR assays verifying the enrichment of PARP10 in NSUN2 RIP assays in SKOV3 cells. (**I**) RT-qPCR detecting the enrichment of PARP10 mRNA in ALYREF RIP assays in SKOV3 cells. (**J**) Detecting protein expression levels in ovarian cancer cells upon NSUN2 knockdown by western blotting assays. (**K**) The expression of the protein was quantified by grayscale in (**J**). (**L**) The diagram of eCLIP experiments of ALYREF in ovarian cancer cells. (**M**) Effect of ALYREF or NSUN2 knockdown on the stability of wild-type or m^{5}C-mutated PARP10 mRNA in ovarian cancer cells. $n = 3$ independent experiments (**B**–**D**, **F**–**I**, **K**, **M**). Data are shown as means ± S.D. *P* value was calculated by one-way ANOVA test with multiple comparisons (**B**, **F**, **K**) or unpaired two-sided Student's *t* test (**C**, **D**, **H**, **I**). Source data are available online for this figure.

 © The Author(s)

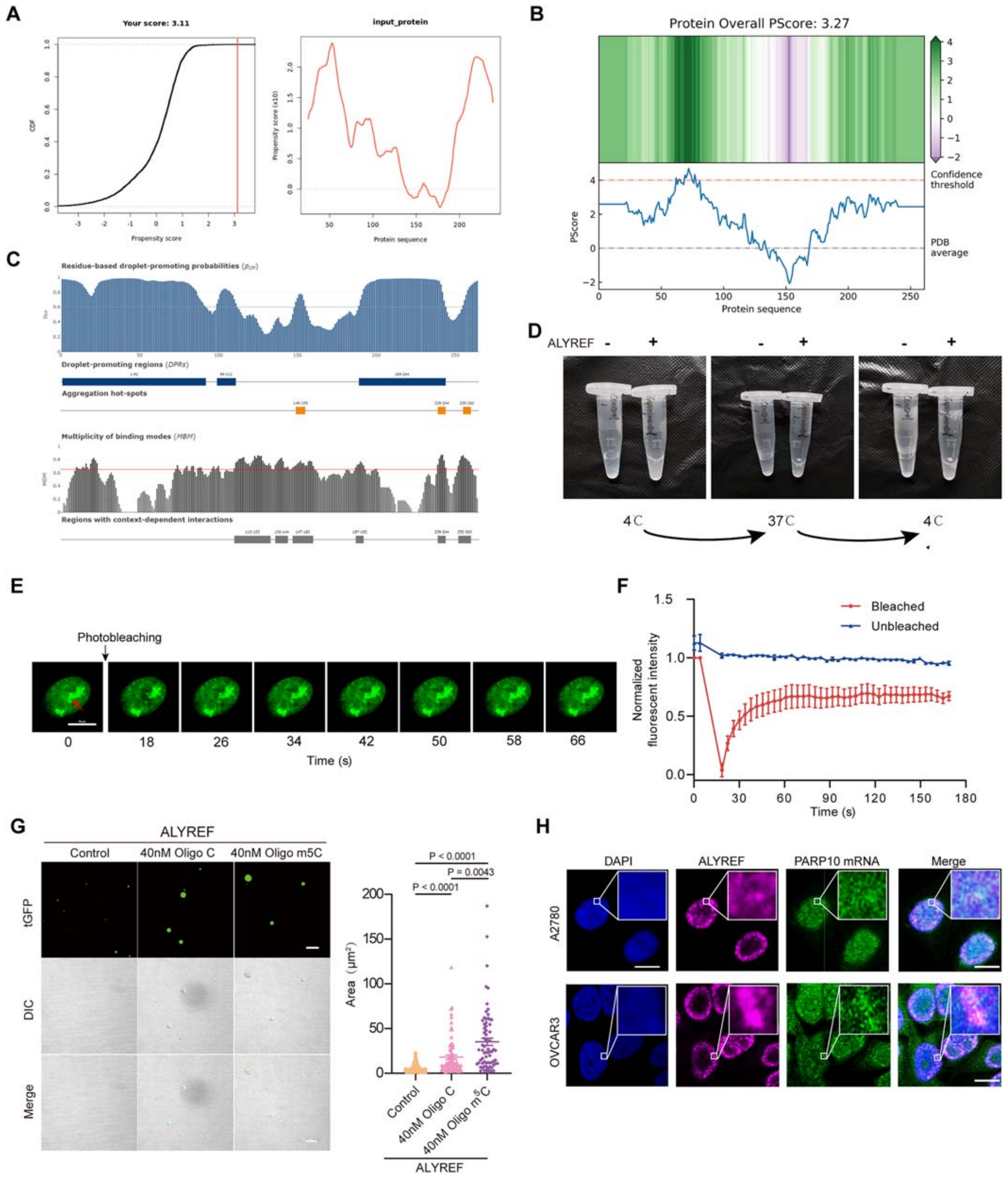
A
Your score: 3.11
CDF
Propensity score
input_protein
Propensity score (x10)
Protein sequence
B
Protein Overall PScore: 3.27
Confidence threshold
PScore
PDB average
Protein sequence
C
Residue-based droplet-promoting probabilities
Droplet-promoting regions (DPRs)
Aggregation hot-spots
Multiplicity of binding modes (MBM)
Regions with context-dependent interactions
D
ALYREF - + - + - +
4℃
37℃
4℃
E
Photobleaching
0
18
26
34
42
50
58
66
Time (s)
F
Bleached
Unbleached
Normalized fluorescent intensity
Time (s)
G
ALYREF
Control
40nM Oligo C
40nM Oligo m5C
tGFP
DIC
Merge
P < 0.0001
P = 0.0043
P < 0.0001
Area（μm²）
Control
40nM Oligo C
40nM Oligo m⁵C
ALYREF
H
DAPI
ALYREF
PARP10 mRNA
Merge
A2780
OVCAR3

© The Author(s)

Figure EV5. ALYREF undergoes liquid-liquid phase separation.

(**A**) Condensation of ALYREF protein was predicted according to catGRANULE. (**B**) Phase separation of ALYREF was predicted by the π Interaction Score Prediction Tool. (**C**) The phase separation of ALYREF was predicted by https://fuzdrop.bio.unipd.it/predictor. (**D**) Effect of temperature on the phase transition properties of ALYREF protein. (**E**) Fluorescence recovery assays after photobleaching of ALYREF condensates in ovarian cancer cells. The red arrow represents the photobleached region in the droplet. Scale bar, 10 µm. (**F**) Quantitative analysis of condensates in (**E**). The black arrow represents the time point of photobleaching. The red arrow represents the photobleached region in the droplet. The red curve represents the average of the normalized fluorescence intensities in different droplet photobleaching regions ($n = 3$ independent experiments). (**G**) Effect of m^5C-modified RNA on phase separation of ALYREF proteins ($n = 50$). Scale bar, 20 µm. (**H**) Co-localization of ALYREF protein and PARP10 RNA by RNA FISH assays in ovarian cancer cells. Scale bar, 10 µm. Data are shown as means ± S.D. *P* value was calculated by one-way ANOVA test with multiple comparisons (**G**). Source data are available online for this figure.

© The Author(s)

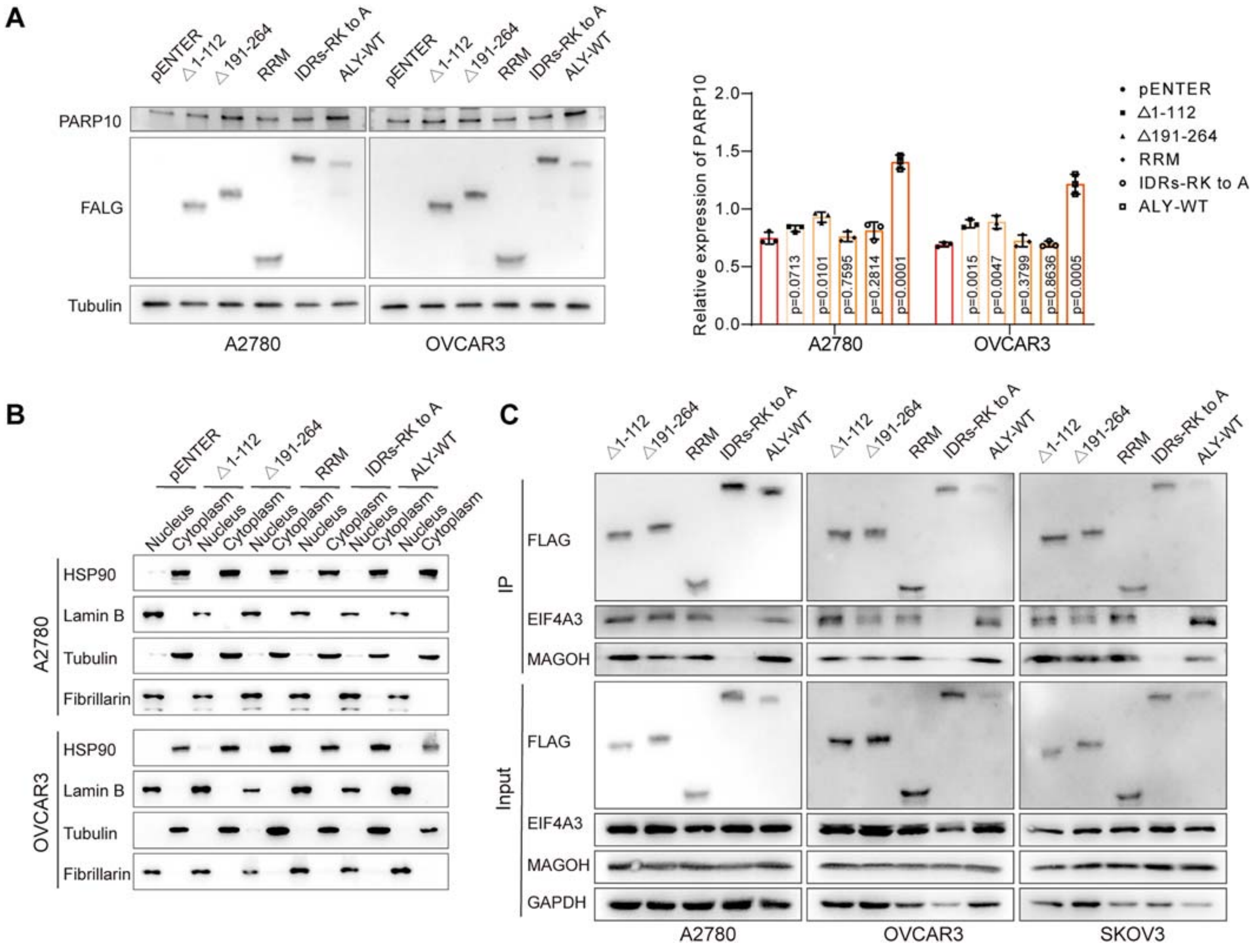

Figure EV6. Interaction of ALYREF with EIF3A and MAGOH.

(**A**) Western blotting verifying the protein of ALYREF-wt, truncations, and mutant expression levels, and quantitative analysis ($n = 3$ independent experiments). Data are shown as means ± S.D. *P* value was calculated by one-way ANOVA test with multiple comparisons. (**B**) Detecting the effectiveness of the nuclear plasma separation assays by western blotting. (**C**) IP assays detecting the interaction of wild-type or mutated ALYREF with EIF3A and MAGOH. Source data are available online for this figure.

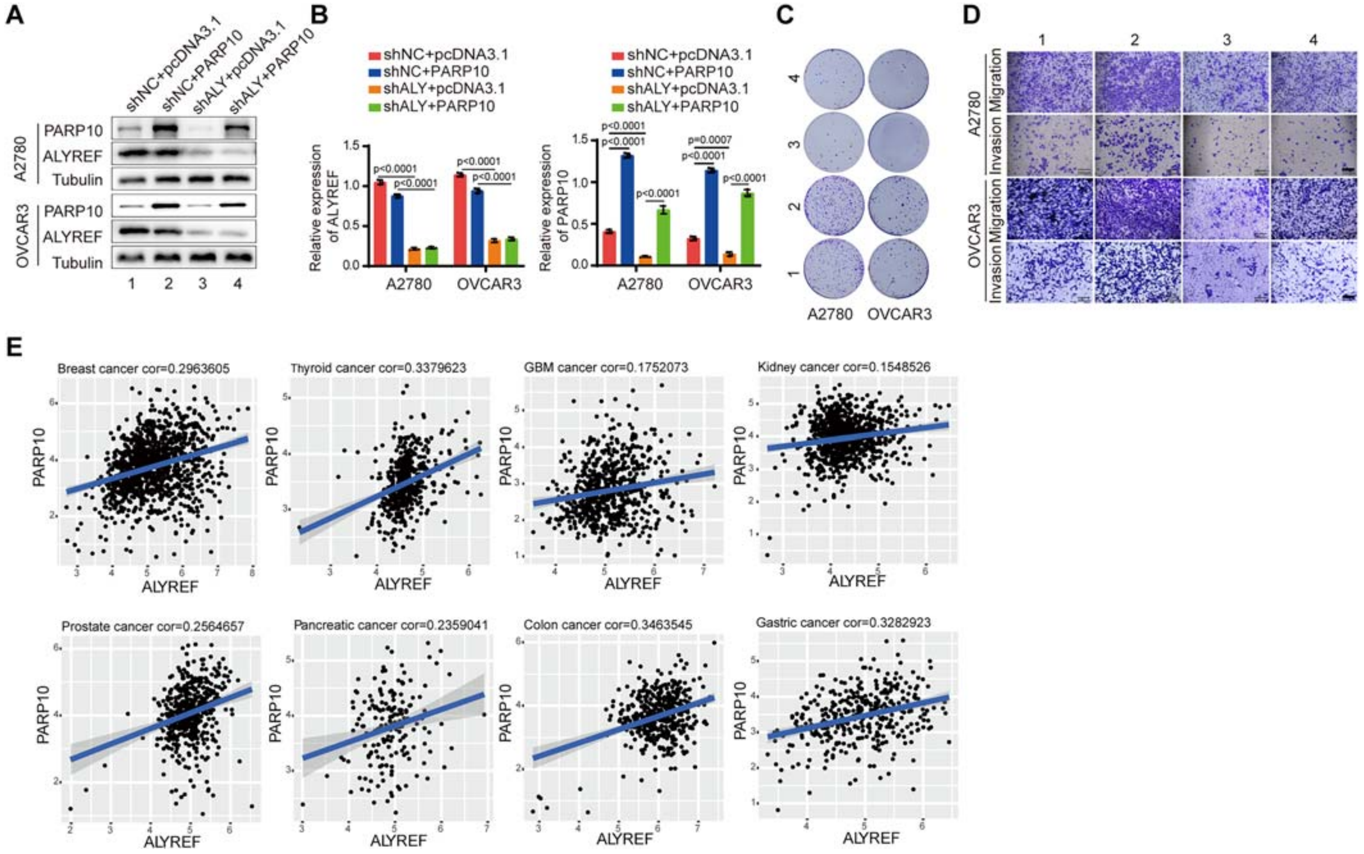

Figure EV7. Key functions of the ALYREF-PARP10 axis in ovarian cancer.

(**A**) Detecting the protein level in ALYREF-depleted ovarian cancer cells with PARP10 overexpression by western blotting. (**B**) The expression of the protein was quantified by grayscale in (**A**) ($n = 3$ independent experiments). Data are shown as means ± S.D. *P* value was calculated by one-way ANOVA test with multiple comparisons. (**C**) Colony formation assays of ALYREF-depleted ovarian cancer cells with overexpression of PARP10. (**D**) The transwell migration and matrigel invasion assays of ALYREF-depleted ovarian cancer cells with overexpression of PARP10. Scale bar, 100 μm. (**E**) Correlation analysis between ALYREF and PARP10 expression in various cancers. Source data are available online for this figure.

 © The Author(s)

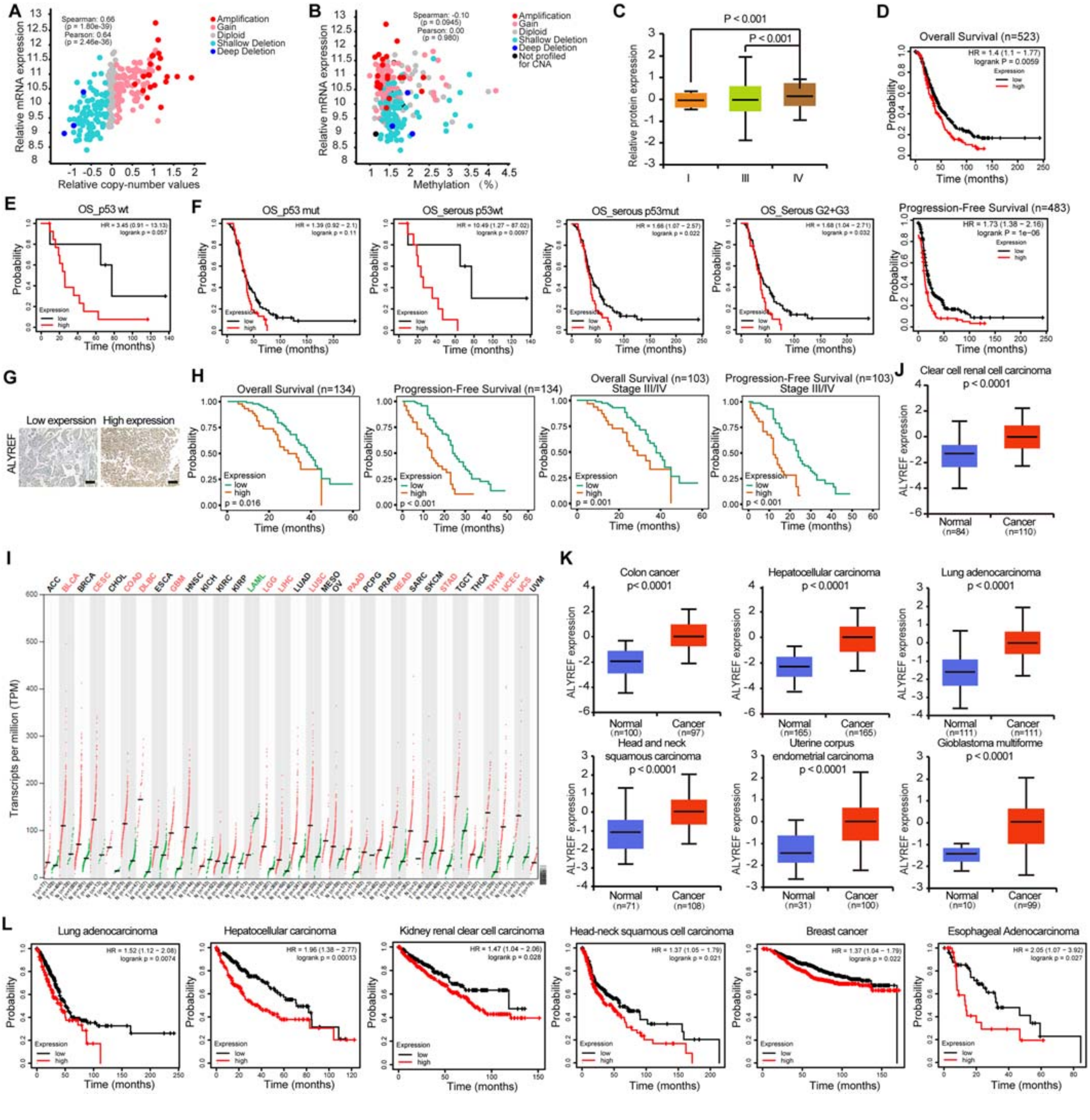

Figure EV8. ALYREF is highly expressed in pan-cancer.

(**A**) Correlation analysis of ALYREF mRNA expression with its gene amplification. (**B**) Correlation analysis of ALYREF mRNA expression with DNA methylation. (**C**) ALYREF expression in serous ovarian cancer was significantly correlated with tumor FIGO stage. (**D**) Survival analysis of ovarian cancer patients with different PARP10 expressions according to the Kaplan-Meier Plotter. (**E**, **F**) Survival analysis of subtype ovarian cancer patients with different ALYREF expressions according to the Kaplan-Meier Plotter. (**G**) Representative IHC images of ALYREF expression in ovarian cancer tissues. Scale bar, 100 μm. (**H**) Survival analysis of ovarian cancer patients with different ALYREF expressions or patients at advanced stage (III and IV) with different ALYREF expressions. (**I–K**) Pan-cancer analysis of ALYREF RNA and protein expression in various cancers according to the TCGA (**I**) and CPTAC databases (**J**, **K**). (**L**) Survival analysis of cancer patients with different ALYREF expressions according to the Kaplan-Meier Plotter. The central line within the box represents the median value (**C**, **J**, **K**). The upper and lower edges of the box represent the 75th and 25th percentiles, respectively. The whiskers extend from the box to the maximum and minimum values within 1.5 times the interquartile range, with any data points beyond this range considered as outliers. *P* value was calculated by two-sided Log-rank (Mantel-Cox) test (**D–F**, **H**, **L**) or unpaired two-sided Student's *t* test (**J**, **K**). Source data are available online for this figure.

© The Author(s)
